# Mechanoresponsive Smad5 Enhances MiR-487a Processing to Promote Vascular Endothelial Proliferation in Response to Disturbed Flow

**DOI:** 10.3389/fcell.2021.647714

**Published:** 2021-04-20

**Authors:** Wei-Li Wang, Li-Jing Chen, Shu-Yi Wei, Yu-Tsung Shih, Yi-Hsuan Huang, Pei-Lin Lee, Chih-I Lee, Mei-Cun Wang, Ding-Yu Lee, Shu Chien, Jeng-Jiann Chiu

**Affiliations:** ^1^Institute of Cellular and System Medicine, National Health Research Institutes, Miaoli, Taiwan; ^2^Departments of Bioengineering and Medicine and Institute of Engineering in Medicine, University of California, San Diego, San Diego, CA, United States; ^3^Department of Biological Science and Technology, China University of Science and Technology, Taipei, Taiwan; ^4^School of Medical Laboratory Science and Biotechnology, College of Medical Science and Technology, Taipei Medical University, Taipei, Taiwan; ^5^Ph.D. Program in Medical Biotechnology, College of Medical Science and Technology, Taipei Medical University, Taipei, Taiwan; ^6^Taipei Heart Institute, Taipei Medical University, Taipei, Taiwan; ^7^Institute of Biomedical Engineering, National Tsing Hua University, Hsinchu, Taiwan; ^8^Institute of Polymer Science and Engineering, National Taiwan University, Taipei, Taiwan

**Keywords:** disturbed flow, endothelium, MicroRNA, shear stress, Smad

## Abstract

MicroRNAs (miRs) and bone morphogenetic protein receptor–specific Smads are mechano-responsive molecules that play vital roles in modulating endothelial cell (EC) functions in response to blood flow. However, the roles of interplay between these molecules in modulating EC functions under flows remain unclear. We elucidated the regulatory roles of the interplay between miR-487a and Smad5 in EC proliferation in response to different flow patterns. Microarray and quantitative RT-PCR showed that disturbed flow with low and oscillatory shear stress (OS, 0.5 ± 4 dynes/cm^2^) upregulates EC miR-487a in comparison to static controls and pulsatile shear stress (12 ± 4 dynes/cm^2^). MiR-487a expression was higher in ECs in the inner curvature (OS region) than the outer curvature of the rat aortic arch and thoracic aorta and also elevated in diseased human coronary arteries. MiR-487a expression was promoted by nuclear phospho-Smad5, which bound to primary-miR-487a to facilitate miR-487a processing. Algorithm prediction and luciferase reporter and argonaute 2-immunoprecipitation assays demonstrated that miR-487a binds to 3′UTR of CREB binding protein (CBP) and p53. Knockdown and overexpression of miR-487a decreased and increased, respectively, phospho-Rb and cyclin A expressions through CBP and p53. A BrdU incorporation assay showed that miR-487a enhanced EC proliferation under OS *in vitro* and in disturbed flow regions of experimentally stenosed rat abdominal aorta *in vivo*. These results demonstrate that disturbed flow with OS induces EC expression of miR-487a through its enhanced processing by activated-Smad5. MiR-487 inhibits its direct targets CBP and p53 to induce EC cycle progression and proliferation. Our findings suggest that EC miR-487 may serve as an important molecular target for intervention against disturbed flow–associated vascular disorders resulting from atherosclerosis.

## Introduction

Endothelial cell (EC) dysfunction is a critical step leading to vascular pathologies, including atherosclerosis, which develop preferentially in arterial branches and curvatures, where the local flow is disturbed with low and oscillatory shear stress (OS) (Chiu and Chien, 2011). In contrast, the straight part of the artery, which is exposed to sustained laminar flow with pulsatile shear stress (PS) having a clear direction, is generally spared from atherosclerotic lesions (Chiu and Chien, 2011). Recent studies show that disturbed flow with OS accelerates EC cycle progression and proliferation (Chiu and Chien, 2011) to promote atherogenesis (Obikane et al., 2010; Chiu and Chien, 2011). Bone morphogenetic protein (BMP)-4 and BMP receptor (BMPR)-specific Smads (i.e., Smad1/5/8) are shown to be activated in ECs by disturbed flow with OS (Sorescu et al., 2003). In our previous studies, we demonstrate that BMPR-specific Smad1/5 can be activated by OS to accelerate EC cycle progression and proliferation *in vitro* and *in vivo* (Zhou et al., 2012). Although BMPR-specific Smads are shown to regulate EC responses to OS, the detailed mechanisms by which BMPR-specific Smads regulate OS-induced EC cycle progression and proliferation remain unclear.

MicroRNAs (MiRs) are noncoding small RNAs, typically 18–22 nucleotides in length, which can regulate gene expression at the posttranscriptional level by interacting with the 3′ untranslated region (3′UTR) of the target message RNA (mRNA) (Bartel, 2009). More than 200 miRs have been found in human ECs, some of which can be regulated by different flow patterns and shear stresses to modulate vascular homeostasis and pathophysiological processes (Donaldson et al., 2018). Laminar shear stress (LS) and PS can upregulate a set of miRs, including miR-23b (Wang et al., 2010), miR-10a (Lee et al., 2017), miR-126 (Mondadori dos Santos et al., 2015), miR-30-5p (Demolli et al., 2015), miR-181b (Sun et al., 2014), miR-143/145 (Kohlstedt et al., 2013; Schmitt et al., 2014), and miR-146a (Chen et al., 2015) in ECs to inhibit their proliferation or inflammation and, hence, atherogenesis (Donaldson et al., 2018). In contrast, OS upregulates miR-92a (Wu et al., 2011), miR-663 (Ni et al., 2011), miR-712 (Son et al., 2013), and miR-21 (Zhou et al., 2011) to promote EC pro-inflammatory responses and atherogenesis. A recent study by Davis et al. (2010) demonstrates that Smads can bind to the consensus sequence, i.e., RNA-Smad binding element (R-SBE: 5′-CAGAC-3′ or 5′-CAGGG-3′) of the primary transcripts of transforming growth factor (TGF)-β/BMP-regulated miRs to promote their processing from primary transcript (pri-miR) to precursor form (pre-miR). Although there have been considerable studies on the roles of miRs in modulating EC responses to fluid flow and shear stress, the molecular mechanisms by which disturbed flow with OS induces the processing of miRs to modulate EC cell cycle and proliferation remain unclear. We postulated that disturbed flow with OS can activate BMPR-specific Smads to regulate the processing of TGF-β/BMP-regulated miRs to subsequently induce EC cycle progression and proliferation.

P53 is a tumor suppressor that plays vital roles in modulating cell cycle and proliferation (Levine, 1997). The relationship between p53 and miRs in vascular physiology and pathophysiology is not clear. It has been shown that p53 activity can be regulated by posttranslational modifications, such as acetylation (Kruse and Gu, 2009). P53 was shown to be acetylated by cAMP-responsive element-binding protein (CREB) (CBP)/p300, which is an acetyltransferase and transcriptional cofactor that serves as a tumor suppressor (Goodman and Smolik, 2000), and CBP/p300-associated factor (pCAF) in response to a variety of cellular stress signals (Brooks and Gu, 2011). The acetylation of p53 can enhance its stability by inhibiting ubiquitination (Li et al., 2002). LS has been shown to induce the upregulation of p53 (Lin et al., 2000) and its acetylation at Lys-382 in ECs (Zeng et al., 2003). Whether OS can modulate p53 expression and acetylation through miRs in ECs to promote their cell cycle progression and proliferation remains unclear.

In the present study, which ranges from *in vitro* cell culture research on effects of different flow patterns and shear stresses on molecular signaling to *in vivo* investigations on the experimentally stenosed rat abdominal aorta and clinical specimens from patients with coronary artery disease (CAD), we demonstrate that disturbed flow with OS induces EC expression of miR-487a, which contains the R-SBE sequence. This OS-induction of miR-487a is regulated by its association with BMPR-specific Smad5, which can promote the processing of pri-miR-487a to pre-miR-487a in EC nuclei. The OS-induction of miR-487a enhances EC proliferation *via* direct targeting of p53 and CBP to increase Rb phosphorylation and cyclin A expression. Our results suggest that miR-487a is a critical molecule for intervention against disturbed flow–induced EC dysfunction and its associated vascular disorders.

## Materials and Methods

### Cell Culture and Flow Apparatus

Human aortic ECs obtained commercially (Clonetics, Palo Alto, CA, United States) (Tsai et al., 2009) were grown in medium 199 (M199; Gibco, Grand Island, NY, United States) supplemented with 20% fetal bovine serum (FBS; Gibco). Cells between passages four and seven were used in the experiments. Cultured ECs were subjected to PS at 12 ± 4 dynes/cm^2^ or OS at 0.5 ± 4 dynes/cm^2^ in a parallel-plate flow chamber as previously described (Lee et al., 2017). Detailed procedures are described in online Supplementary Documents.

### RNA-Immunoprecipitation (IP) Assay

ECs subjected to flow or transfected with control miR or pre-miR-487a were lysed in a lysis buffer containing protease inhibitor cocktail (Schmitt et al., 2014). Detailed procedures are described in online Supplementary Documents.

### Animal Model of Aortic Stenosis

A U-shaped titanium clip (Ethicon Endo-Surgery) was surgically applied to the rat abdominal aorta as previously described (Zhou et al., 2012). The animal experiments were performed in accordance with National Institutes of Health guidelines and with the approval of the Animal Research Committee of National Health Research Institutes. Detailed procedures are described in online Supplementary Documents.

### En Face Staining

The formalin-fixed rat aorta tissues were washed with Tris-buffered saline (TBS). The aorta was longitudinally dissected with micro-dissecting scissors and pinned flat for *en face* staining as previously described (de Planell-Saguer et al., 2010). Detailed procedures are described in online Supplementary Documents.

### RNA-electrophoretic mobility shift assay

RNA-electrophoretic mobility shift assay (EMSA) was conducted by using a bio-UTP-labeled pri-miR-487a probe (∼150 nt). The pri-miR-487a probe was heat-denatured at 65°C for 6 min and renatured prior to being added to the binding buffer containing EC nuclear proteins (10 μg). Detailed procedures are described in online Supplementary Documents.

### Pri-miR-487a Processing Assay

The pri-miR-487a processing assay was performed as previously described (Guil and Caceres, 2007). In brief, the biotin-labeled, full-length pri-miR-487a containing 522 nucleotides was prepared by *in vitro* transcription with T7 RNA polymerase in the presence of biotin-UTP, using a human miR-487a gene cloned into a pcDNA3.1(+) vector as a template. Detailed procedures are described in online Supplementary Documents.

### Generation of Luciferase Reporter Constructs

To generate reporter vectors bearing miR-487a binding sites and wild-type and mutant 3′UTR of CBP and p53, the sense and antisense strands of oligonucleotides bearing the predicted miR-487a binding sequences were commercially synthesized, annealed, and cloned into HindIII and MluI of the pMIR-REPORT luciferase vector (Ambion). Detailed procedures are described in online Supplementary Documents.

### Statistics Analysis

Results are expressed as mean ± SEM. Statistical significance was determined using the Mann-Whitney rank sum test in the program SigmaStat 3.5 for two groups of data and a one-way analysis of variance (ANOVA), followed by Scheffe’s test for multiple comparisons. The level of statistical significance was defined as *P* < 0.05 from three to five separate experiments.

An expanded Methods section is available in the online Supplementary Documents.

## Results

### OS Induces miR-487a Expression in ECs *in vitro*

**FIGURE 1:**
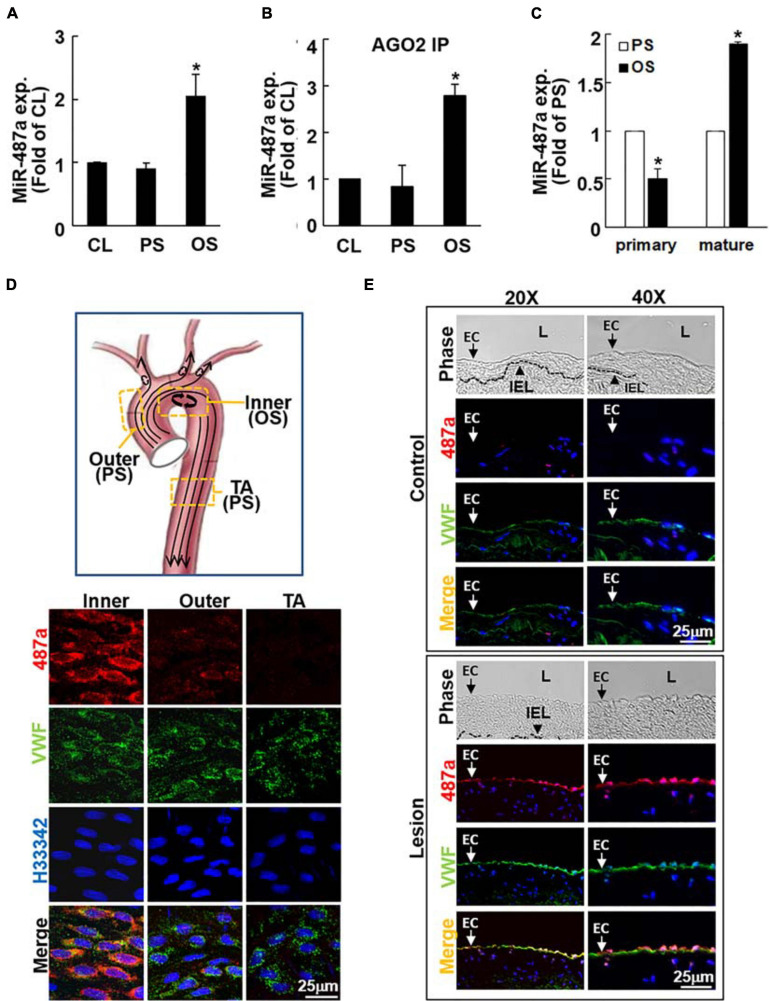
OS induces mature miR-487a expression in ECs *in vitro* and *in vivo*. ECs were kept under static conditions as controls (CL) or exposed to OS or PS for 24 h, and the mature **(A)** Ago2-associated mature **(B)** and primary **(C)** miR-487a expressions of EC lysates were analyzed by quantitative RT-PCR. Data are mean ± SEM from three independent experiments. *P < 0.05 vs. static or PS-stimulated cells. **(D)** The inner and outer curvatures of aortic arch and the straight segment of thoracic aorta of normal rats were examined by *en face* immunostaining for miR-487a and vWF with H33342 nuclear counterstains. **(E)** Serial cross-sections of diseased human coronary arteries were stained for human miR-487a and vWF and counterstained with H33342. IEL, internal elastic lamina. L, lumen. Arrow denotes the EC layer. Results are representative of five independent experiments with similar results.

Because our previous studies demonstrate that BMPR-specific Smads can be activated by OS in ECs (Zhou et al., 2012) and that Smads may promote maturation of miRs containing Smad binding elements (Davis et al., 2008, 2010), we first identified the OS-responsive miRs whose expression could be regulated by BMPR-specific Smads in ECs. Three independent groups of experiments, i.e., ECs under static conditions and exposed to OS (0.5 ± 4 dynes/cm^2^) or PS (12 ± 4 dynes/cm^2^) for 24 h, were performed with TaqMan^®^ Human MicroRNA array A, which contains 380 human miR probes. The results showed that nine of 380 human miRs are significantly upregulated by OS in ECs in comparison to PS-stimulated and static control cells (Table 1 and online Supplementary Table 1). Among these miRs, miR-487a was most upregulated by OS (2.15-fold increase, *P* < 0.05) and contains the putative Smad binding elements (Table 1). This OS-induced miR-487a expression in ECs was confirmed by quantitative RT-PCR analysis of these cells (Figure 1A). Because the targeting of mRNA by an miR is dependent on its association with Argonaute (Ago) protein to form functional miR-induced silencing complex (miRISC) (Bartel., 2009), we investigated whether OS induces the association of Ago2 with miR-487a in ECs. The Ago2-IP assay showed that miR-487a is significantly enriched in the Ago2-containing miRISC in OS-stimulated ECs, as compared with PS-stimulated and static control cells (Figure 1B). Contrary to the mature miR-487a, the expression of pri-miR-487a was decreased in ECs under OS stimulation in comparison to PS (Figure 1C). These results indicate that OS promotes the progression of miR-487a from primary to mature form in ECs.

**TABLE 1:** Disturbed flow–responsive miRs in ECs.

MiR names	Mean fold change (OS/CL)	*p-*value	Mean fold change (PS/CL)	*p-*value	RNA-Smad binding element
hsa-miR-487a	2.15	0.03	1.42	0.77	aaucauacagggacauccaguu
has-miR-92a	2.00	0.02	0.69	0.01	
hsa-miR-576	1.84	0.04	3.27	0.52	–
has-miR-21	1.6	<0.01	2.14	<0.01	uagcuuaucagacugauguuga
hsa-miR-181a	1.43	0.05	1.18	0.55	–
hsa-miR-125a-3p	1.34	0.04	2.27	0.04	
hsa-miR-181c	1.33	0.04	0.69	0.29	–
hsa-let-7g	1.27	0.05	0.94	0.75	–
hsa-let-7a	1.20	<0.01	1.05	0.79	–

*ECs were kept under static conditions as controls (CL) or exposed to OS (0.5 ± 4 dynes/cm^2^) or PS (12 ± 4 dynes/cm^2^) for 24 h, and their total RNAs were collected and subjected to miR microarray analysis. Data represent triplicate experiments with similar results. Primary miR-487a and miR-21 contain RNA-Smad binding element sequence in their stem regions, where the Smad proteins are able to bind to regulate miR processing at the post-transcriptional level.*

### MiR-487a Is Upregulated in ECs in Athero-Susceptible, Disturbed Flow Regions of the Native Circulation and Diseased Human Coronary Arteries *in vivo*

To investigate whether the OS-induction of miR-487a found in cultured ECs *in vitro* also exists in the native circulation *in vivo*, we examined the aortic arch and the straight segment of thoracic aorta of normal rats by *en face in situ* hybridization of miR-487a and immunostaining for von Willebrand factor (vWF), with H33342 nuclear counterstaining. The results show that high levels of miR-487a expression are present in the EC cytoplasm in the inner curvature of aortic arch, where disturbed flow with OS occurs (Chiu and Chien, 2011), but not in the outer curvature and the straight segment of the thoracic aorta, where laminar flow with PS prevails (Chiu and Chien, 2011; Figure 1D). To further assess miR-487a expression in atherosclerotic lesions, immunohistochemical examinations were made on diseased human coronary arteries with the use of internal thoracic arteries from the same patients as controls. The results showed that miR-487a is highly expressed in the EC layer (indicated by arrows) but not in the neointima in the lesion region (Figure 1E). In contrast, the control thoracic aorta showed virtually no detectable staining of miR-487a in the EC layer. These results demonstrate that EC miR-487a is specifically induced in athero-susceptible, disturbed flow regions in the native circulation *in vivo* and in diseased human coronary arteries. Thus, the OS-induction of EC miR-487a may play important roles in the formation and progression of atherosclerosis.

### OS Induces Smad5 Activation in ECs to Promote miR-487a Processing

**FIGURE 2:**
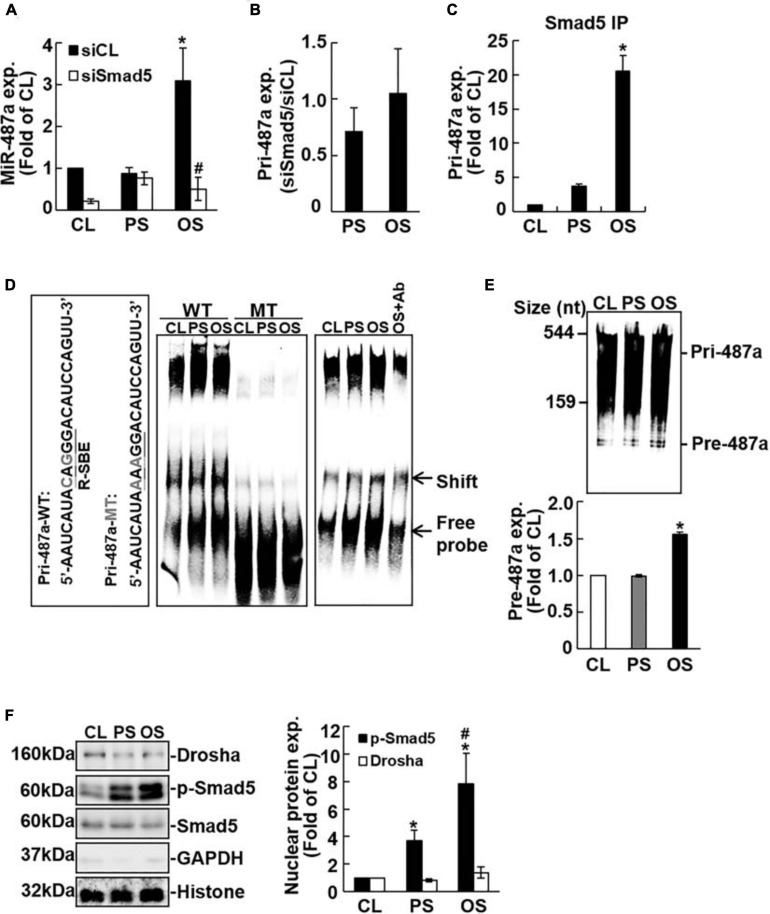
Processing of EC pri-miR-487a is promoted by its association with nuclear phospho-Smad5 in response to OS. ECs were transfected with control (siCL) or Smad5-specific siRNA (siSmad5) (40 nM each) for 48 h before exposure to flow. The expression of the mature **(A)** primary **(B)** miR-487a and pri-miR-487a in Smad-immunoprecipitated complex **(C)** were analyzed by quantitative RT-PCR. Data are mean ± SEM from three independent experiments. *P < 0.05 vs. static control cells. ^#^*P* < 0.05 vs. siCL. **(D)** The nuclear fraction of conditioned ECs was extracted and subjected to EMSA using biotin-labeled WT or R-SBE mutant (mut) pri-miR-487a as the probe. **(E)** *In vitro* pri-miR-487a processing assay was performed by incubating nuclear extracts with biotin-labeled pri-miR-487a substrate. Results in panels **(D**,**E)** are representative of triplicate experiments with similar results. **(F)** Nuclear and cytosol localizations of the designated proteins were detected by Western blot analysis. Data are mean ± SEM from three independent experiments. *P < 0.05 vs. static control cells. ^#^*P* < 0.05 vs. PS-stimulated cells.

It has been reported that Smad proteins may not affect the transcription of miRs, but they may bind to Drosha and R-SBE of primary miRs to promote miR processing (Davis et al., 2008, 2010). Transfecting ECs with Smad5-specific siRNA abolished OS-induction of mature miR-487a (Figure 2A) with no significant influences on primary miR-487a expression in these ECs (Figure 2B). Moreover, application of shear stress to ECs did not affect Drosha expression in these ECs (Figure 2F). These results indicate that the OS-induction of EC mature miR-487a is attributable to the binding of Smad5 to primary miR-487a to promote miR-487a processing, but not due to the modulations in primary miR-487a and Drosha expression levels in these OS-stimulated cells.

To test this hypothesis, RNA-IP analysis was performed to investigate whether Smad5 can assemble with pri-miR-487a in ECs under flow. As shown in Figure 2C, application of OS to ECs for 24 h induced association of Smad5 with pri-miR-487a in these ECs as compared with static and PS-stimulated cells. We further investigated whether the R-SBE sequence of miR-487a is essential for the recruitment of Smad5. A 2-bp mutation was introduced into the R-SBE of pri-miR-487a (pri-miR-487a-mut), and the interaction between Smad5 and pri-miR-487a was examined by RNA electrophoretic mobility shift assay (RNA-EMSA) with pri-miR-487a-mut and pri-miR-487a-WT (wild type) as probes. The results show that use of the pri-miR-487a-WT probe resulted in the formation of RNA-protein complex, which was inhibited by R-SBE mutation (shift in Figure 2D). Application of OS to ECs for 24 h resulted in increases in the RNA-protein complex formation in ECs as compared with static and PS-stimulated cells (Figure 2D). This OS-induction of RNA-Smad5 protein complex was confirmed by the decrease in RNA-protein complex formation with the use of anti-Smad5 antibody (Figure 2D). To test whether OS can promote pre-miR-487a production, an *in vitro* pri-miR processing assay was performed by incubating biotin-labeled pri-miR487a substrates (544 nucleotides) with nuclear extracts from ECs subjected to OS, PS, or in a static condition. The results show that OS induced increases in pre-miR-487a expression in ECs in comparison to static and PS-stimulated cells, indicating that OS stimuli can facilitate the processing of pri-miR-487a in ECs (Figure 2E). We further used the fractionation assay to investigate whether OS induces Smad5 activation to interact with pri-miR-487a in the EC nuclei. The results show that application of OS to ECs results in significant induction of Smad5 phosphorylation in EC nuclei as compared with static and PS-stimulated cells (Figure 2F). The levels of Drosha proteins are not significantly different in ECs subjected to OS, PS, and the static condition. Taken together, these results indicate that application of OS to ECs induces Smad5 activation and its binding to the R-SBE of pri-miR-487a with Drosha in EC nuclei, which can consequently promote miR-487a processing in these ECs.

### MiR-487a Inhibits CBP and p53 Expressions by Targeting Their 3′UTR

**FIGURE 3:**
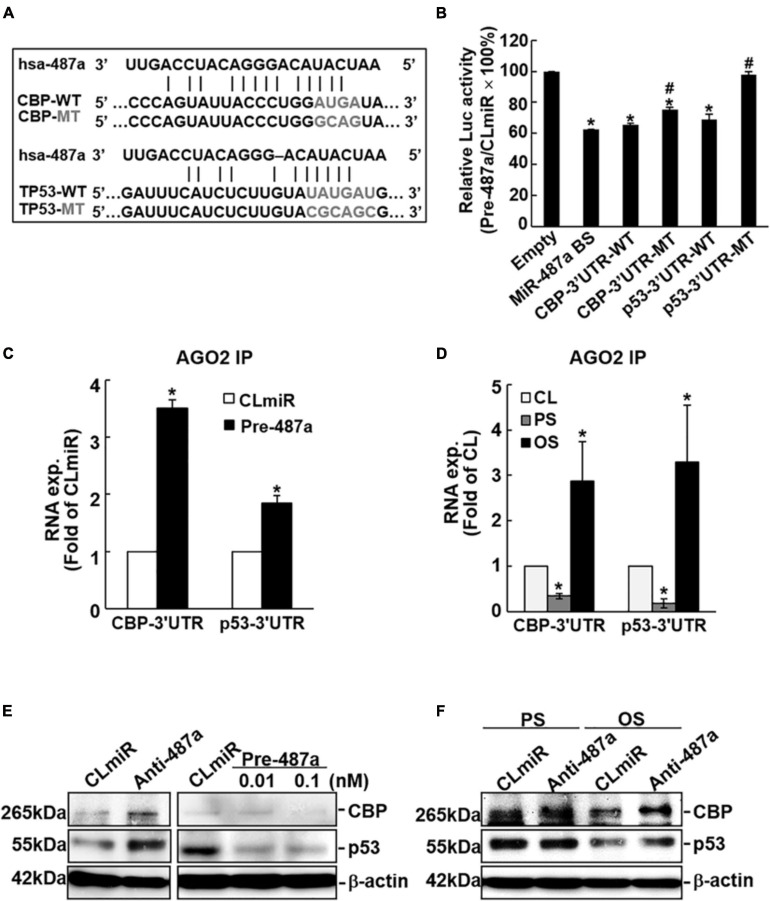
MiR-487a targets 3′UTR of CBP and p53 to regulate their expressions. **(A)** The 3′UTRs of CBP and p53 genes contain miR-487a binding sites. **(B)** A series of pMIR-REPORT luciferase reporter plasmids, including empty vector only, consensus miR-487a binding sequences, wild-type and mutant 3′UTR of CBP and p53, were co-transfected with pre-miR-487a (Pre-487a) or control miR (CLmiR) into ECs for 48 h, and their luciferase (Luc) activity was measured and normalized to β-gal activity. **(C–F)** ECs were transfected with pre-miR-487a, anti-miR-487a (Anti-487a), or CLmiR. In some experiments, ECs were kept as static controls (CL) or exposed to OS or PS for 24 h. Ago2 pull-down assay was performed, and the transcription levels of CBP and p53 in Ago2-immunocomplexes were determined by quantitative RT-PCT **(C,D)**. Protein expressions of CBP and p53 were determined by Western blot analysis **(E,F)**. Data represent triplicate experiments with similar results. *P < 0.05 vs. static control. Data in panels **(B,C,D)** are mean ± SEM from three independent experiments. Results in panels **(E,F)** are representative of triplicate experiments with similar results. *P < 0.05 vs. empty vector **(B)**, CLmiR **(C)**, or static control cells **(D)**. ^#^*P* < 0.05 vs. wild-type 3′UTR.

The use of bioinformatics databases miRanda, RNAhybrid, miRNAMap, and miRWALK predicted that human CBP and p53 are potential targets of miR-487a. Both CBP and p53 contain miR-487a binding sites in the 3′UTRs of their mRNAs (Figure 3A). To investigate whether miR-487a can directly target CBP and p53, we constructed a series of luciferase reporter plasmids containing predicted miR-487a recognition sequences in the 3′-UTRs of CBP and p53. The sequence that is a direct match of the miR-487a binding site was used as a positive control. Together with pre-miR-487a or a negative control RNA, these constructed reporter plasmids were transfected into ECs. The results show that transfection with pre-miR-487a decreases the luciferase activity of the reporter vectors with an miR-487a binding site, CBP-3′UTR, and p53-3′UTR to 0. 63-, 0.66, and 0.69-fold, respectively, as compared with the control vector (Figure 3B). Mutations of the miR-487a binding site in CBP-3′UTR and p53-3′UTR returned the luciferase activity levels to 0.75 (*P* < 0.05) and 0.98 (*P* < 0.01) folds, respectively. The Ago2-immunoprecipitation assay showed enrichments of CBP- and p53-3′UTRs in miRISCs in ECs transfected with pre-miR-487a (Figure 3C) or subjected to OS (Figure 3D). Overexpression of pre-miR-487a in ECs under static conditions inhibited CBP and p53 protein expressions in these ECs (Figure 3E). In contrast, transfection of ECs with anti-miR-487a increased CBP and p53 protein expressions in these ECs (Figure 3E). Application of OS to ECs for 24 h resulted in inhibitions in CBP and p53 expressions in ECs (Figure 3F). These OS-inhibitions in CBP and p53 expressions were rescued by transfecting ECs with anti-miR-487a. Taken together, our results indicate that both CBP and p53 are direct target molecules of miR-487a and that OS inhibits CBP and p53 expressions *via* upregulating miR-487a in ECs.

### OS Induces Rb Phosphorylation and Cyclin A Expression Through Upregulating miR-487a and Downregulating CBP and p53 Expressions in ECs

**FIGURE 4:**
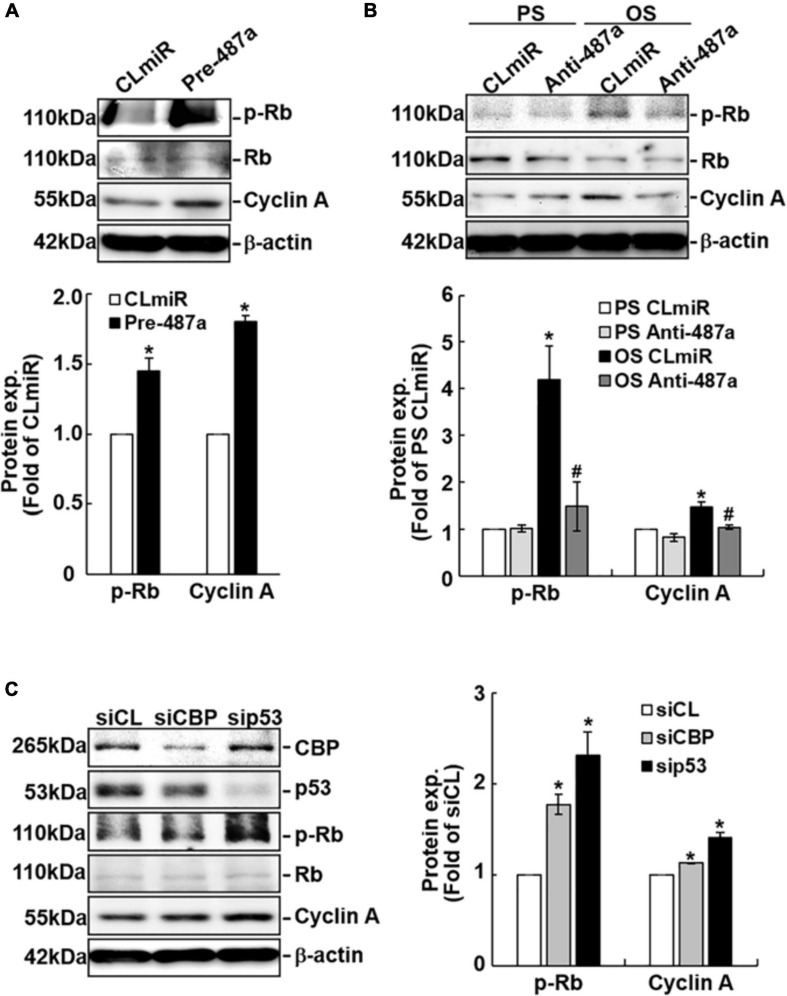
MiR-487a regulates Rb phosphorylation and cyclin A expression through CBP and p53 in ECs. ECs were transfected with control miR (CLmiR), pre-miR-487a (Pre-487a) **(A)**, or anti-miR-487a (Anti-487a) **(B)** for 48 h, and then kept under static conditions (CL) or exposed to PS or OS. In some experiments, ECs were transfected with CBP- and p53-specific siRNAs (siCBP and sip53) **(C)** for 48 h, and then kept under static conditions as controls. Cell lysates were collected, and the cell cycle regulatory protein expressions were determined by Western blot analysis. Data are mean ± SEM from three independent experiments. *P < 0.05 vs. CLmiR **(A)**, PS CLmiR **(B)**, or siCL **(C)**. ^#^*P* < 0.05 vs. OS CLmiR **(B)**.

To investigate whether cell cycle regulatory proteins are regulated by miR-487a in ECs in response to flow, ECs were transfected with pre-miR-487a or anti-miR-487a and then kept under the static condition or subjected to different flow patterns. Transfecting ECs with pre-miR-487a under the static condition induced Rb phosphorylation and cyclin A expression (Figure 4A). Application of OS to ECs for 24 h also induced these cell cycle regulatory proteins (Figure 4B). These OS-induced responses were inhibited by transfecting ECs with anti-miR-487a, indicating that OS induces Rb phosphorylation and cyclin A expression through upregulating miR-487a in ECs. Moreover, knockdown of CBP and p53 in ECs by their specific siRNAs resulted in increases in Rb phosphorylation and cyclin A expression in ECs (Figure 4C). Taken together, our data show that OS-induction of miR-487a downregulates CBP and p53 expressions to upregulate phospho-Rb and cyclin A, which may consequently promote cell cycle progression in ECs.

**FIGURE 5:**
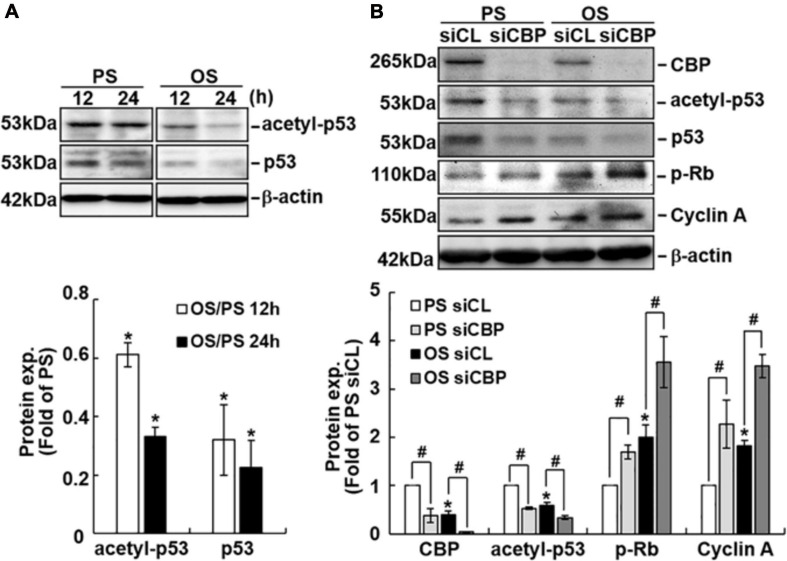
CBP acetylates p53 in ECs in response to PS. ECs were subjected to OS vs. PS for the indicated times **(A)**. In some experiments, ECs were transfected with control (siCL) or CBP-specific siRNA (siCBP) for 48 h before subjecting to flows **(B)**. Protein expression was detected by Western blot analysis using the indicated antibodies. Data are mean ± SEM from three independent experiments. *P < 0.05 vs. PS stimuli **(A)** or PS and siCL **(B)**. ^#^*P* < 0.05 vs. siCL **(B)**.

Because CBP has been shown to acetylate and, hence, stabilize p53 (Brooks and Gu, 2011) and our data show that knockdown of CBP decreases the protein levels of p53 (Figure 4C), we investigated whether different flow patterns exert differential effects on EC p53 acetylation to regulate its expression. Exposure of ECs to OS for 12 or 24 h caused lower expression levels of acetylated-p53 and p53 proteins (Figure 5A) and higher levels of phospho-Rb and cyclin A proteins (Figure 5B) in these ECs in comparison to PS-stimulated cells. Knockdown of CBP by its specific siRNAs resulted in downregulation of acetylated-p53 and upregulation of phospho-Rb and cyclin A expressions in both PS- and OS-stimulated ECs (Figure 5B). These results suggest that the OS-mediated decreases in p53 expression in ECs may be attributable, at least in part, to the decreased expression of CBP, which resulted in reduction of p53 acetylation in ECs in response to OS.

### MiR-487a Plays Important Roles in Modulating EC Proliferation in Response to OS *in vivo* and *in vitro*

**FIGURE 6:**
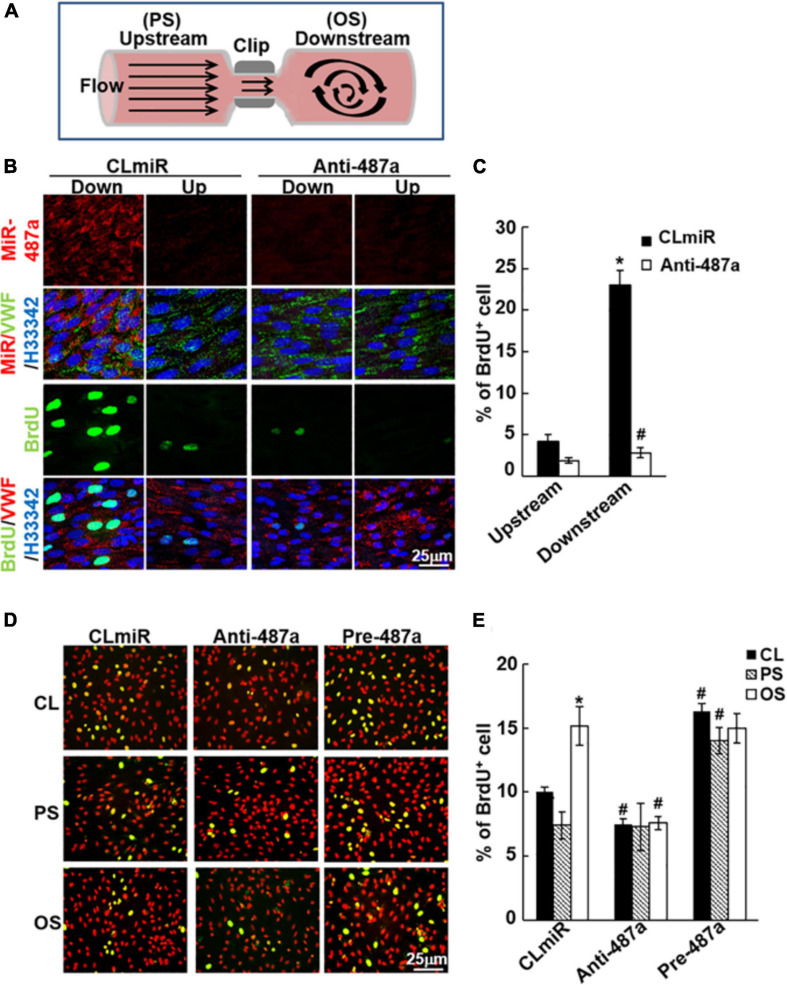
MiR-487a enhances EC proliferation in response to OS *in vivo* and *in vitro*. **(A)** Schematic diagram showing the creation of stenosis in the rat abdominal aorta by using a U-shaped clip. **(B)** Anti-miR-487a or control saline (*n* = 5 each) was mixed with invivofectamine (Invitrogen) and injected into the rat abdominal aorta that had been subjected to stenosis using a U-clip. BrdU was injected i.v. into the rats 1 week after surgery and 1 day before sacrifice. *En face* staining of miR-487a, BrdU, vWF, and H33342 was performed on the luminal surfaces of the affected aortas. **(C)** Statistic data showing that anti-miR-487a administration inhibits disturbed flow-induced BrdU uptake in ECs in regions downstream to constricted areas. Data are mean ± SEM from three independent experiments. *P < 0.05 vs. upstream area. ^#^*P* < 0.05 vs. CLmiR. **(D,E)** ECs were transfected with anti-miR-487a (Anti-487a) or pre-miR-487a (Pre-487a) for 48 h and then were kept as static controls or exposed to OS or PS. BrdU incorporation assay was performed. Data are mean ± SEM from three independent experiments. *P < 0.05 vs. static controls. ^#^*P* < 0.05 vs. CLmiR.

To assess whether miR-487a is involved in OS-induced EC proliferation *in vivo*, we used a stenosis model in which the rat abdominal aorta was subjected to partial constriction by using a U-clip (Figure 6A; Zhou et al., 2012). This placement of the U-clip resulted in a 65% constriction of the aorta diameter, which induced an accelerated forward pulsatile flow pattern in the constricted region, followed by a pronounced oscillating flow with the existence of retrograde velocities in the downstream region of poststenotic dilatation (Zhou et al., 2012). Anti-miR-487a or control miR was locally injected into the affected aorta during 40 min of vascular occlusion and then removed before flow resumption. The cell proliferation marker bromodeoxyuridin (BrdU) was administered intravenously 24 h before sacrifice. *En face* immunostaining showed that miR-487a was highly expressed in ECs in the downstream OS region in control animals (Figure 6B). This upregulation of EC miR-487a in the downstream OS region was accompanied by significant increases in the BrdU uptake as compared with the upstream PS region (Figures 6B,C). *In vivo* administration with anti-miR-487a resulted in inhibitions in miR-487a expression and BrdU uptake in ECs in the downstream OS region. This miR-487a-mediated EC proliferation induced by disturbed flow with OS *in vivo* was substantiated by our *in vitro* flow assay, which shows that applications of OS and PS to ECs for 24 h result in upregulation and downregulation of BrdU uptake in ECs in comparison to control cells, respectively (Figures 6D,E). Transfecting ECs with anti-miR-487a inhibited OS-induced BrdU uptake in ECs, whereas overexpression of pre-miR-487a increased BrdU uptake in PS-stimulated and control cells. Taken together, our results indicate that miR-487a plays important roles in modulating EC proliferation in response to OS *in vivo* and *in vitro*.

## Discussion

**FIGURE 7:**
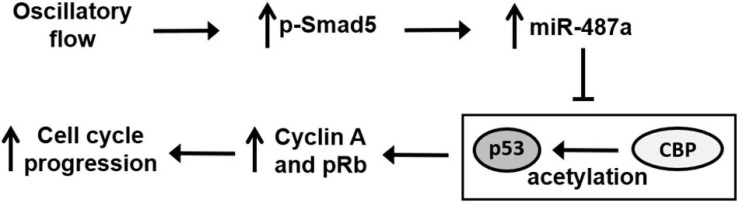
Schematic diagram of miR-487a signaling and its modulation in gene expression and function in ECs in response to OS. OS activates Smad5 to promote miR-487a processing, which directly targets the 3′UTR of CBP and p53 to repress their expressions, thus inhibiting their anti-proliferative function through the inhibition in Rb phosphorylation and cyclin A expression, leading to cell cycle progression and proliferation.

This study has identified EC miR-487a as a novel mechanoresponsive miR, whose expression can be induced by disturbed flow with OS to promote EC proliferation *in vitro* and *in vivo*. This disturbed flow–induction of miR-487a was mediated by BMPR-specific Smad5, which promotes miR-487a processing in ECs in response to disturbed flow. Several lines of evidence support this conclusion (summarized in Figure 7). First, exposure of ECs to OS but not PS induced miR-487a expression in ECs with increased enrichment of miR-487a in the Ago2-containing miRISC. This OS-induction of miR-487a was also observed in the EC layer of athero-susceptible, disturbed flow regions in rat aortic arch *in vivo* and diseased human coronary arteries. Second, application of OS to ECs induced activation of Smad5, which bound to the R-SBE of primary miR-487a in EC nuclei to promote the processing and maturation of miR-487a. Third, miR-487a can bind to the 3′UTR of CBP and p53 to inhibit their expressions in ECs in response to OS. These OS-inhibitions of CBP and p53 expressions resulted in increases in Rb phosphorylation and cyclin A expression in ECs. Fourth, application of OS to ECs resulted in decreases in acetylation levels of p53. These responses were attributable, at least in part, to the OS-reduction of CBP, which is shown to acetylate and, hence, stabilize p53. Finally, knockdown of miR-487a by its antagomir inhibited OS-induced EC proliferation *in vitro* and *in vivo*, indicating the important roles of miR-487a in promoting EC proliferation in response to OS. Thus, our findings provide new insights into the mechanisms by which disturbed flow with OS activates Smad protein to induce the processing and maturation of miR-487a in ECs, which can directly target CBP and p53 to induce Rb phosphorylation and cyclin A expression with the consequent promotion of EC proliferation.

MiRs have been shown to play pivotal roles in modulating signaling, gene expression, and functions in ECs and, hence, vascular biology and pathobiology in health and diseases (Donaldson et al., 2018). There is accumulating evidence that different flow patterns and shear stresses may play differential roles in modulating miR expression in ECs with the consequent modulation in EC function and atherogenesis. Although laminar or pulsatile flow with PS upregulates a set of EC miRs, including miR-23b (Wang et al., 2010), miR-10a (Lee et al., 2017), miR-126 (Mondadori dos Santos et al., 2015), miR-30-5p (Demolli et al., 2015), miR-181b (Sun et al., 2014), miR-143/145 (Kohlstedt et al., 2013; Schmitt et al., 2014), and miR-146a (Chen et al., 2015) to inhibit EC proliferation or inflammation, disturbed flow with OS upregulates miR-92a (Wu et al., 2011), miR-663 (Ni et al., 2011), miR-712 (Son et al., 2013), and miR-21 (Zhou et al., 2011) to promote EC pro-inflammatory responses and atherogenesis. In the present study, we demonstrated for the first time that miR-487a is a novel mechanoresponsive miR in ECs that can be induced by disturbed flow with OS. This disturbed flow–induction of EC miR-487a is attributable to the promotion of miR-487a processing but not primary miR-487a expression in ECs in response to disturbed flow. Several lines of evidence support this notion. First, application of OS to ECs reduced the expression of primary miR-487a in ECs. Second, mature miR-487a was significantly enriched in the Ago2-containing miRISC in OS-stimulated ECs, indicating that OS induces the expression of functional miR487a in ECs. Third, the *in vitro* pri-miR processing assay, in which biotin-labeled pri-miR487a substrates are incubated with nuclear extracts from sheared ECs, demonstrated that OS induces increases in EC pre-miR-487a expression. Fourth, *en face* immunostaining using an LNA-miR-487a probe to detect the mature form of miRs *in situ* (Nuovo, 2008) showed that mature miR-487a is highly expressed in the EC cytoplasm in the disturbed flow region of the rat aortic arch and experimentally stenosed abdominal aorta *in vivo*. Thus, our findings indicate that disturbed flow induces the processing and maturation, but not transcription, of miR-487a in ECs.

Smad proteins (Davis et al., 2008) and other cofactors, including KSRP (Trabucchi et al., 2009), FUS/TLS (Morlando et al., 2012), and YAP (Mori et al., 2014) are shown to be able to associate with microprocessor proteins Drosha and DGCR8 to regulate the processing of miRs in cells (Gregory et al., 2006; Han et al., 2006). Our previous studies demonstrate that disturbed flow with OS induces the activation of BMPR-specific Smad1/5 in ECs to contribute to their cell cycle progression and proliferation and, hence, atherogenesis (Zhou et al., 2012). Because miR-487a contains the putative RNA-Smad binding elements within the stem region of the primary transcript (Davis et al., 2010), we hypothesized that this OS-activation of BMPR-specific Smads may participate in the OS-induced processing of miR-487a in ECs. This hypothesis was confirmed by the following findings. First, knockdown of Smad5 abolished the OS-induction of mature miR-487a but had no effect on primary miR-487a expression in ECs. Second, the RNA-IP assay demonstrated that OS induces association of Smad5 with pri-miR-487a in ECs. Third, Smad5-IP, RNA-EMSA, and *in vitro* processing assays demonstrated that Smad5 can bind to the R-SBE element of pri-miR-487a to induce its processing in ECs in response to OS. It is known that primary miRs are transcribed and processed to precursor miRs in the nucleus (Kim et al., 2009) and that Smads’ activation upon TGF-β/BMP stimulation is accompanied by their nuclear translocation to reach the regulatory targets (Chen and Xu, 2011). In the present study using a nuclear fractionation assay found that the levels of phospho-Smad5, but not Drosha, in the nucleus are much higher in ECs subjected to OS stimulation as compared with static control and PS-stimulated ECs. This fractionation assay further demonstrated that application of OS to ECs results in an increase in Smad5-pri-miR-487a complex formation in EC nuclei in comparison to static and PS-stimulated cells. Thus, our findings indicate that disturbed flow can activate Smad5 and increase its localization to EC nuclei to enhance its binding to pri-miR-487a, thereby promoting miR-487a processing in ECs.

As shown in Table 1, miR-21 is also upregulated by OS and has the RNA-Smad binding element. This result is in agreement with the results of our previous study, which shows that the transcriptional level of miR-21 could be upregulated by disturbed flow with OS *via* the c-Jun signaling pathway in ECs (Zhou et al., 2011). In addition, Smad1/5 are shown to modulate miR-21 processing in human pulmonary arterial smooth muscle cells in response to BMPs (Davis et al., 2008, 2010). Thus, it is possible that disturbed flow with OS could regulate the processing of miR-21 in ECs *via* the activated Smad5. However, Weber et al. (2010) found that application of prolonged unidirectional shear stress (15 dynes/cm^2^) to ECs for 24 h could upregulate miR-21 expression in these ECs. These results implicate that the mechanisms underlying shear stress–regulated miR-21 expression in ECs are complicated and warrant further investigation.

Recent studies show that miR-487a is upregulated in several types of tumor cells to regulate genes involved in tumor progression, including sprouty-related EVH1 domain containing two (Chang et al., 2017), phosphoinositide-3-kinase regulatory subunit 1 (Chang et al., 2017), breast cancer resistance protein (Ma et al., 2013), and membrane-associated guanylate kinase inverted 2 (Ma et al., 2016). However, the role of miR-487a and its molecular mechanisms in modulating EC functions and vascular biology remained unclear. Our present study provides the first evidence that miR-487a can regulate cell cycle regulatory proteins in ECs and their proliferation in response to OS. *In silico* analysis predicted that miR-487a can directly target CBP and p53 in cells. The present study on ECs has validated this bioinformatics prediction by using the luciferase reporter and Ago2-IP assays to show that miR-487a can bind to the 3′UTR of CBP and p53 in ECs and that CBP- and p53-3′UTRs are enriched in miRISCs in ECs overexpressing miR-487a or subjected to OS. Knockdown and overexpression of miR-487a resulted in upregulation and downregulation of CBP and p53 in ECs, respectively. Moreover, subjection of ECs to OS resulted in downregulation of CBP and p53 in comparison to PS-stimulated ECs. This OS-mediated downregulation of CBP and p53 in ECs can be rescued by knockdown of miR-487a. The regulatory roles of miR-487a in EC cell cycle and proliferation through CBP and p53 were further substantiated by their regulations of Rb phosphorylation and cyclin A expression in ECs. Overexpression of miR-487a and knockdowns of CBP and p53 resulted in increases in Rb phosphorylation and cyclin A expression in ECs. Taken together, our data provide new insights into the mechanisms by which disturbed flow with OS induces EC cycle progression and proliferation by directly targeting CBP and p53 and their regulations in Rb phosphorylation and cyclin A expression in ECs.

The acetylation level of p53 is correlated to its activation and stabilization (Brooks and Gu, 2011). Zeng et al. (2003) found that laminar flow with LS increases the level of acetylation of p53 at lysine 382, which is recognized as the CBP/p300 acetylation site (Zeng et al., 2003). In the present study, we found that application of OS to ECs reduces the level of acetylation of p53 at lysine 382 as compared with PS-stimulated cells. Knockdown of CBP resulted in decreases in p53 acetylation at lysine 382 and its expression in ECs. These findings suggest that OS-reduction of p53 expression in ECs may be attributable, at least in part, to the downregulation of CBP, which leads to decreases in p53 acetylation and, hence, its expression in OS-stimulated ECs.

The present study has the following physiological and pathophysiological significance. We have characterized miR-487a as an important mechanoresponsive molecule that connects the chain of events of disturbed flow, mechanical sensing, and EC cycle progression and proliferation in the arterial wall. We also present evidence that the role of miR-487a in modulating OS-induced EC proliferation is mediated through its regulation of CBP and p53 expressions and downstream Rb phosphorylation and cyclin A expression. Our findings that OS induces bindings of Smad5 to the R-SBE element of pri-miR-487a to increase the Smad5-pri-miR-487a complex formation in EC nuclei indicate that OS induces the processing and maturation of miR-487a in ECs. In concert with the previous findings that the miR-487a level is upregulated in serum of patients with atypical coronary artery disease in comparison to healthy controls (Wang et al., 2014), our present data that miR-487a is highly expressed in the EC layer of athero-susceptible regions of the rat aortic arch and abdominal stenosed aorta and diseased human coronary arteries implicate that EC miR-487a may play important roles in the formation and progression of atherosclerosis.

In summary, the present study used a combination of cell culture, experimental animals, and clinical specimens to demonstrate that endothelial miR-487a expression is induced by disturbed flow with OS both *in vitro* and *in vivo* (including the endothelial layers of rat aorta subjected to different flow patterns and diseased human coronary arterial wall) with consequent promotion of EC proliferation. Our findings suggest that endothelial miR-487a may be a promising molecular target for therapeutic intervention against vascular disorders associated with disturbed flow-induced EC dysfunction, such as atherosclerosis.

## Data Availability Statement

The datasets presented in this study can be found in online repositories. The names of the repository/repositories and accession number(s) can be found in the article/Supplementary Material.

## Ethics Statement

The studies involving human participants were reviewed and approved by The Hospital Human Subjects Review Committee and Ethics Review Board of the National Health Research Institutes (Number: EC1020901-E). The patients/participants provided their written informed consent to participate in this study. The animal study was reviewed and approved by National Institutes of Health guidelines and with the approval of the Animal Research Committee of National Health Research Institutes.

## Author Contributions

W-LW, L-JC, S-YW, Y-TS, Y-HH, P-LL, C-IL, M-CW carried out experimental work. W-LW, J-JC designed experiments. W-LW, D-YL, SC, and J-JC participated in discussion and wrote manuscript. J-JC supervised research work. All authors contributed to the article and approved the submitted version.

## Conflict of Interest

The authors declare that the research was conducted in the absence of any commercial or financial relationships that could be construed as a potential conflict of interest.
