# Supplementary material for: Mechanoresponsive Smad5 Enhances MiR-487a Processing to Promote Vascular Endothelial Proliferation in Response to Disturbed Flow
Source: Front Cell Dev Biol. 2021 Apr 20;9:647714. doi: 10.3389/fcell.2021.647714 (PMC8093806; doi:10.3389/fcell.2021.647714)
Supplement: Supplementary file 1 [file Table_1.DOCX]

**Online Table S1. Microarray analysis of miR expression in ECs in response to different flows.**

| **MiR names** | **Mean fold change**  **(OS/CL)** | ***p*-value** | **Mean fold change**  **(PS/CL)** | ***p*-value** |
| --- | --- | --- | --- | --- |
| **hsa-miR-487a** | ***2.15 ± 0.28** | **0.03** | **1.42 ± 1.08** | **0.77** |
| **hsa-miR-92a** | ***2.00 ± 0.25** | **0.02** | **0.69 ± 0.06** | **0.01** |
| **hsa-miR-576** | ***1.84 ± 0.24** | **0.04** | **3.27 ± 2.62** | **0.52** |
| **hsa-miR-21** | ***^,^**1.6 ± 0.08** | **< 0.01** | **2.14 ± 0.14** | **< 0.01** |
| **hsa-miR-181a** | ***1.43 ± 0.13** | **0.05** | **1.18 ± 0.23** | **0.55** |
| **hsa-miR-125a-3p** | ***^,^**1.34 ± 0.09** | **0.04** | **2.27 ± 0.36** | **0.04** |
| **hsa-miR-181c** | ***1.33 ± 0.09** | **0.04** | **0.69 ± 0.20** | **0.29** |
| **hsa-let-7g** | ***1.27 ± 0.08** | **0.05** | **0.94 ± 0.13** | **0.75** |
| **hsa-let-7a** | ***1.20 ± 0.02** | **< 0.01** | **1.05 ± 0.15** | **0.79** |
| **hsa-miR-124** | ****1.61 ± 0.40** | **0.28** | **6.02 ± 0.70** | **< 0.01** |
| **hsa-miR-145** | ****0.72 ± 0.07** | **0.01** | **2.76 ± 0.36** | **0.01** |
| **hsa-miR-23b** | ****1.1 ± 0.16** | **0.57** | **2.43 ± 0.27** | **0.01** |
| **hsa-miR-10a** | ****0.43 ± 0.05** | **< 0.01** | **2.38 ± 0.28** | **0.01** |
| **hsa-miR-19a** | ****1.09 ± 0.17** | **0.63** | **2.06 ± 0.36** | **0.04** |
| **hsa-miR-143** | ****1.53 ±0.33** | **0.25** | **1.90 ± 0.19** | **0.02** |
| **has-miR-155** | ****0.93 ± 0.03** | **0.01** | **1.88 ± 0.16** | **0.01** |
| **hsa-miR-101** | ****1.10 ± 0.17** | **0.58** | **1.45 ± 0.11** | **0.01** |
| **hsa-miR-502-5p** | **12.45 ± 9.83** | **0.51** | **24.21 ± 19.14** | **0.50** |
| **hsa-miR-486-3p** | **9.14 ± 3.87** | **0.16** | **7.86 ± 5.11** | **0.33** |
| **hsa-miR-451** | **8.21 ± 5.17** | **0.44** | **3.49 ± 2.73** | **0.60** |
| **hsa-miR-449a** | **7.76 ± 4.43** | **0.28** | **1.18 ± 0.53** | **0.79** |
| **hsa-miR-32** | **7.06 ± 4.74** | **0.36** | **1.86 ± 0.77** | **0.41** |
| **hsa-miR-508-3p** | **6.02 ± 4.54** | **0.53** | **N/A** |  |
| **hsa-miR-330-5p** | **5.93 ± 3.14** | **0.40** | **4.07 ± 2.78** | **0.53** |
| **hsa-miR-518e** | **5.63 ± 2.31** | **0.29** | **5.95 ± 4.31** | **0.52** |
| **hsa-miR-523** | **5.55 ± 3.40** | **0.34** | **5.30 ± 3.56** | **0.38** |
| **hsa-miR-885-5p** | **4.75 ± 1.46** | **0.10** | **4.90 ± 2.59** | **0.29** |
| **hsa-miR-342-5p** | **4.68 ± 3.51** | **0.44** | **0.34 ± 0.03** | **0.00** |
| **hsa-miR-331-5p** | **3.92 ± 1.34** | **0.26** | **19.17 ± 15.14** | **0.50** |
| **hsa-miR-200c** | **3.64 ± 0.80** | **0.13** | **8.68 ± 6.53** | **0.51** |
| **hsa-miR-326** | **3.45 ±1.94** | **0.36** | **1.46 ± 0.41** | **0.42** |
| **hsa-miR-548b-5p** | **3.29 ± 1.83** | **0.49** | **2.62 ± 1.13** | **0.43** |
| **hsa-miR-199b-5p** | **2.84 ± 1.99** | **0.60** | **7.84 ± 4.14** | **0.37** |
| **hsa-miR-202** | **2.71 ± 0.62** | **0.09** | **1.24 ± 0.54** | **0.74** |
| **hsa-miR-542-5p** | **2.42 ± 1.70** | **0.64** | **5.83 ± 4.28** | **0.53** |
| **hsa-miR-423-5p** | **2.41 ± 0.68** | **0.16** | **2.14 ± 0.44** | **0.10** |
| **hsa-miR-190** | **2.40 ± 0.94** | **0.29** | **10.17 ± 6.18** | **0.29** |
| **hsa-miR-422a** | **2.38 ± 0.67** | **0.17** | **1.14 ± 0.47** | **0.81** |
| **hsa-miR-296-5p** | **2.31 ± 0.68** | **0.19** | **1.62 ± 0.46** | **0.33** |
| **hsa-miR-133a** | **2.29 ± 1.40** | **0.50** | **0.19 ± 0.03** | **0.00** |
| **hsa-miR-455-3p** | **2.10 ± 0.70** | **0.27** | **1.66 ± 0.77** | **0.52** |
| **hsa-miR-218** | **2.03 ± 0.57** | **0.22** | **1.22 ± 0.20** | **0.42** |
| **hsa-miR-98** | **1.88 ± 0.60** | **0.29** | **1.22 ± 0.15** | **0.31** |
| **hsa-miR-654-5p** | **1.86 ± 0.27** | **0.06** | **0.66 ± 0.49** | **0.60** |
| **hsa-miR-887** | **1.78 ± 0.82** | **0.48** | **0.79 ± 0.35** | **0.64** |
| **hsa-miR-629** | **1.76 ± 1.00** | **0.57** | **0.52 ± 0.11** | **0.02** |
| **hsa-miR-362-3p** | **1.70 ± 0.37** | **0.20** | **1.41 ± 0.92** | **0.73** |
| **hsa-miR-655** | **1.65 ± 0.49** | **0.34** | **1.00 ± 0.37** | **1.00** |
| **hsa-miR-137** | **1.65 ±0.63** | **0.45** | **0.15 ± 0.09** | **0.00** |
| **hsa-miR-502-3p** | **1.54 ± 0.49** | **0.42** | **1.40 ± 0.13** | **0.06** |
| **hsa-miR-29b** | **1.50 ± 0.44** | **0.41** | **1.23 ± 0.12** | **0.20** |
| **hsa-miR-27a** | **1.48 ± 0.20** | **0.12** | **1.19 ± 0.21** | **0.51** |
| **hsa-miR-203** | **1.48 ±0.31** | **0.27** | **2.12 ± 0.77** | **0.30** |
| **hsa-miR-628-5p** | **1.45 ± 0.35** | **0.35** | **1.28 ± 0.32** | **0.51** |
| **hsa-miR-23a** | **1.44 ± 0.16** | **0.09** | **1.25 ± 0.28** | **0.51** |
| **hsa-miR-362-5p** | **1.41 ± 0.59** | **0.60** | **0.82 ± 0.09** | **0.18** |
| **hsa-miR-519a** | **1.39 ± 0.42** | **0.50** | **2.07 ± 0.95** | **0.41** |
| **hsa-miR-486-5p** | **1.38 ± 0.72** | **0.69** | **0.42 ± 0.15** | **0.04** |
| **hsa-miR-518b** | **1.36 ± 0.56** | **0.62** | **2.49 ± 1.40** | **0.43** |
| **hsa-miR-185** | **1.35 ± 0.27** | **0.35** | **1.15 ± 0.29** | **0.69** |
| **hsa-miR-369-5p** | **1.35 ±0.66** | **0.69** | **0.93 ± 0.37** | **0.88** |
| **hsa-miR-542-3p** | **1.35 ± 0.33** | **0.43** | **0.68 ± 0.41** | **0.56** |
| **hsa-miR-301b** | **1.31 ± 0.18** | **0.24** | **1.00 ± 0.38** | **1.00** |
| **hsa-miR-335** | **1.30 ± 0.29** | **0.45** | **1.11 ± 0.56** | **0.88** |
| **hsa-miR-193a-3p** | **1.29 ± 0.22** | **0.34** | **1.45 ± 0.26** | **0.23** |
| **hsa-miR-216b** | **1.29 ± 0.40** | **0.58** | **1.06 ± 0.19** | **0.81** |
| **hsa-miR-224** | **1.29 ± 0.29** | **0.46** | **0.96 ± 0.07** | **0.63** |
| **hsa-miR-150** | **1.28 ± 0.12** | **0.13** | **0.78 ± 0.15** | **0.31** |
| **hsa-miR-579** | **1.27 ± 0.43** | **0.64** | **1.65 ± 0.61** | **0.43** |
| **hsa-miR-212** | **1.26 ± 0.28** | **0.49** | **1.40 ± 0.40** | **0.46** |
| **hsa-miR-148a** | **0.85 ± 0.20** | **0.00** | **1.23 ± 0.12** | **0.13** |
| **hsa-miR-29a** | **1.25 ± 0.22** | **0.40** | **1.08 ± 0.12** | **0.62** |
| **hsa-miR-361-5p** | **1.25 ± 0.17** | **0.29** | **1.32 ± 0.37** | **0.51** |
| **hsa-miR-324-5p** | **1.24 ± 0.10** | **0.12** | **1.21 ± 0.14** | **0.29** |
| **hsa-miR-29c** | **1.24 ± 0.22** | **0.43** | **1.01 ± 0.17** | **0.96** |
| **hsa-miR-625** | **1.24 ± 0.47** | **0.70** | **0.97 ± 0.09** | **0.79** |
| **hsa-let-7f** | **1.23 ± 0.19** | **0.38** | **0.86 ± 0.29** | **0.71** |
| **hsa-miR-148b** | **1.22 ± 0.43** | **0.70** | **1.08 ± 0.08** | **0.45** |
| **hsa-miR-886-3p** | **1.21 ± 0.19** | **0.41** | **2.54 ± 0.68** | **0.14** |
| **hsa-miR-31** | **1.2 ± 0.27** | **0.57** | **1.11 ± 0.09** | **0.35** |
| **hsa-miR-376a** | **1.18 ± 0.19** | **0.48** | **0.74 ± 0.06** | **0.03** |
| **hsa-miR-26b** | **1.17 ± 0.17** | **0.47** | **0.91 ± 0.19** | **0.73** |
| **hsa-miR-582-5p** | **1.16 ± 0.20** | **0.64** | **1.37 ± 0.89** | **0.56** |
| **hsa-miR-301a** | **1.16 ± 0.19** | **0.54** | **0.68 ± 0.20** | **0.26** |
| **hsa-miR-199a-3p** | **1.15 ± 0.23** | **0.62** | **0.78 ± 0.21** | **0.45** |
| **hsa-miR-340** | **1.15 ± 0.20** | **0.56** | **0.77 ± 0.07** | **0.06** |
| **hsa-miR-452** | **1.14 ± 0.18** | **0.58** | **0.88 ± 0.21** | **0.66** |
| **hsa-miR-744** | **1.14 ± 0.18** | **0.57** | **1.13 ± 0.08** | **0.25** |
| **hsa-miR-425** | **1.13 ± 0.38** | **0.79** | **0.72 ± 0.11** | **0.11** |
| **hsa-miR-374a** | **1.13 ± 0.29** | **0.73** | **0.78 ±0.13** | **0.23** |
| **hsa-miR-204** | **1.12 ± 0.16** | **0.56** | **0.88 ± 0.07** | **0.25** |
| **hsa-miR-660** | **1.12 ± 0.23** | **0.68** | **1.20 ± 0.19** | **0.45** |
| **hsa-miR-140-5p** | **1.12 ± 0.18** | **0.62** | **0.89 ± 0.11** | **0.48** |
| **hsa-miR-18a** | **1.11 ± 0.20** | **0.67** | **0.79 ± 0.11** | **0.21** |
| **hsa-miR-200b** | **1.11 ± 0.16** | **0.59** | **0.48 ± 0.17** | **0.07** |
| **hsa-miR-125b** | **1.11 ± 0.12** | **0.51** | **0.94 ± 0.16** | **0.76** |
| **hsa-miR-410** | **1.11 ± 0.12** | **0.49** | **0.89 ± 0.18** | **0.64** |
| **hsa-miR-142-3p** | **1.10 ± 0.52** | **0.88** | **0.53 ± 0.15** | **0.06** |
| **hsa-miR-216a** | **1.10 ± 0.12** | **0.50** | **0.95 ± 0.29** | **0.89** |
| **hsa-miR-652** | **1.10 ± 0.12** | **0.52** | **1.05 ± 0.11** | **0.71** |
| **hsa-miR-598** | **1.10 ± 0.18** | **0.67** | **0.72 ± 0.24** | **0.40** |
| **hsa-miR-15b** | **1.09 ± 0.18** | **0.69** | **0.79 ± 0.21** | **0.47** |
| **hsa-miR-18b** | **1.09 ± 0.27** | **0.79** | **0.70 ± 0.12** | **0.11** |
| **hsa-miR-328** | **1.09 ± 0.13** | **0.59** | **0.94 ± 0.14** | **0.72** |
| **hsa-miR-324-3p** | **1.08 ± 0.06** | **0.34** | **1.33 ± 0.18** | **0.22** |
| **hsa-miR-194** | **1.08 ± 0.22** | **0.79** | **0.91 ± 0.27** | **0.79** |
| **hsa-miR-19b** | **1.08 ± 0.18** | **0.74** | **0.76 ± 0.10** | **0.12** |
| **hsa-miR-545** | **1.07 ± 0.13** | **0.70** | **0.23 ± 0.04** | **0.00** |
| **hsa-miR-409-5p** | **1.06 ± 0.21** | **0.84** | **0.68 ± 0.48** | **0.61** |
| **hsa-miR-342-3p** | **1.05 ± 0.23** | **0.86** | **0.77 ± 0.03** | **0.00** |
| **hsa-miR-106a** | **1.05 ± 0.24** | **0.87** | **0.78 ± 0.05** | **0.03** |
| **hsa-miR-106b** | **1.04 ± 0.18** | **0.85** | **0.85 ± 0.13** | **0.40** |
| **hsa-miR-195** | **1.04 ± 0.14** | **0.81** | **0.90 ± 0.12** | **0.55** |
| **hsa-miR-22** | **1.04 ± 0.10** | **0.73** | **1.07 ± 0.25** | **0.84** |
| **hsa-miR-483-5p** | **1.04 ± 0.40** | **0.93** | **1.20 ± 0.31** | **0.63** |
| **hsa-miR-130a** | **1.02 ± 0.17** | **0.92** | **1.04 ± 0.17** | **0.87** |
| **hsa-miR-193a-5p** | **1.02 ± 0.10** | **0.90** | **1.19 ± 0.25** | **0.58** |
| **hsa-miR-17** | **1.01 ± 0.21** | **0.96** | **0.85 ± 0.08** | **0.19** |
| **hsa-miR-93** | **1.01 ± 0.05** | **0.89** | **0.76 ± 0.05** | **0.01** |
| **hsa-miR-20a** | **1.01 ± 0.12** | **0.96** | **0.85 ± 0.08** | **0.19** |
| **hsa-miR-28-5p** | **1.00 ± 0.20** | **0.99** | **0.84 ± 0.10** | **0.27** |
| **hsa-miR-374b** | **1.00 ± 0.09** | **0.98** | **0.67 ± 0.13** | **0.11** |
| **hsa-let-7b** | **1.00 ± 0.04** | **0.94** | **1.00 ± 0.20** | **0.99** |
| **hsa-miR-489** | **0.99 ± 0.41** | **0.99** | **0.88 ± 0.34** | **0.79** |
| **hsa-miR-103** | **0.99 ± 0.12** | **0.97** | **0.90 ± 0.11** | **0.49** |
| **hsa-let-7e** | **0.99 ± 0.04** | **0.80** | **0.89 ± 0.10** | **0.42** |
| **hsa-miR-210** | **0.99 ± 0.13** | **0.94** | **1.33 ± 0.25** | **0.33** |
| **hsa-miR-500** | **0.99 ± 0.20** | **0.96** | **0.91 ± 0.21** | **0.74** |
| **hsa-miR-758** | **0.99 ± 0.19** | **0.96** | **0.51 ± 0.16** | **0.07** |
| **hsa-miR-24** | **0.98 ± 0.13** | **0.91** | **1.15 ± 0.12** | **0.36** |
| **hsa-miR-132** | **0.98 ± 0.06** | **0.78** | **1.15 ± 0.25** | **0.65** |
| **hsa-let-7d** | **0.97 ± 0.12** | **0.87** | **0.92 ± 0.08** | **0.47** |
| **hsa-miR-337-5p** | **0.97 ± 0.18** | **0.91** | **1.20 ± 0.12** | **0.25** |
| **hsa-miR-339-5p** | **0.97 ± 0.18** | **0.91** | **1.12 ± 0.10** | **0.38** |
| **hsa-miR-411** | **0.97 ± 0.16** | **0.90** | **0.65 ± 0.10** | **0.05** |
| **hsa-miR-874** | **0.97 ± 0.12** | **0.85** | **0.98 ± 0.31** | **0.96** |
| **hsa-miR-636** | **0.97 ± 0.25** | **0.93** | **0.79 ± 0.17** | **0.36** |
| **hsa-miR-484** | **0.97 ± 0.08** | **0.76** | **1.05 ± 0.07** | **0.61** |
| **hsa-miR-376c** | **0.97 ± 0.19** | **0.90** | **0.70 ± 0.07** | **0.03** |
| **hsa-miR-15a** | **0.96 ± 0.22** | **0.90** | **1.06 ± 0.21** | **0.83** |
| **hsa-miR-192** | **0.96 ± 0.03** | **0.42** | **1.13 ± 0.13** | **0.47** |
| **hsa-miR-424** | **0.96 ± 0.17** | **0.85** | **0.78 ± 0.17** | **0.35** |
| **hsa-miR-146a** | **0.96 ± 0.26** | **0.91** | **0.97 ± 0.26** | **0.93** |
| **hsa-miR-299-5p** | **0.96 ± 0.33** | **0.93** | **0.64 ± 0.25** | **0.30** |
| **hsa-miR-539** | **0.96 ± 0.22** | **0.88** | **0.82 ± 0.12** | **0.28** |
| **hsa-miR-99b** | **0.96 ± 0.04** | **0.43** | **0.95 ± 0.03** | **0.24** |
| **hsa-miR-454** | **0.95 ± 0.17** | **0.81** | **0.85 ± 0.13** | **0.40** |
| **hsa-miR-26a** | **0.95 ± 0.09** | **0.66** | **0.85 ± 0.09** | **0.25** |
| **hsa-miR-99a** | **0.95 ± 0.08** | **0.61** | **0.86 ± 0.06** | **0.12** |
| **hsa-miR-449b** | **0.93 ± 0.06** | **0.38** | **1.60 ± 0.18** | **0.05** |
| **hsa-miR-16** | **0.93 ± 0.12** | **0.66** | **0.78 ± 0.10** | **0.15** |
| **hsa-miR-25** | **0.93 ± 0.07** | **0.46** | **0.73 ± 0.03** | **0.00** |
| **hsa-miR-323-3p** | **0.93 ± 0.21** | **0.80** | **0.74 ± 0.08** | **0.06** |
| **hsa-miR-382** | **0.93 ± 0.22** | **0.80** | **0.83 ± 0.07** | **0.12** |
| **hsa-miR-95** | **0.93 ± 0.09** | **0.55** | **0.84 ± 0.10** | **0.23** |
| **hsa-miR-186** | **0.93 ± 0.17** | **0.74** | **0.77 ± 0.06** | **0.03** |
| **hsa-miR-126** | **0.92 ± 0.05** | **0.17** | **1.09 ± 0.06** | **0.23** |
| **hsa-let-7c** | **0.92 ± 0.09** | **0.51** | **0.87 ± 0.07** | **0.18** |
| **hsa-miR-130b** | **0.92 ± 0.15** | **0.67** | **0.76 ± 0.04** | **0.01** |
| **hsa-miR-27b** | **0.92 ± 0.07** | **0.38** | **0.69 ± 0.15** | **0.17** |
| **hsa-miR-494** | **0.91 ± 0.05** | **0.21** | **1.24 ± 0.11** | **0.14** |
| **hsa-miR-331-3p** | **0.91 ± 0.20** | **0.74** | **0.88 ± 0.05** | **0.10** |
| **hsa-miR-886-5p** | **0.90 ± 0.09** | **0.42** | **1.20 ± 0.13** | **0.28** |
| **hsa-miR-365** | **0.89 ± 0.12** | **0.52** | **0.71 ± 0.13** | **0.15** |
| **hsa-miR-10b** | **0.89 ± 0.09** | **0.39** | **0.80 ± 0.07** | **0.09** |
| **hsa-miR-532-5p** | **0.89 ± 0.12** | **0.51** | **1.10 ± 0.06** | **0.27** |
| **hsa-miR-590-5p** | **0.89 ± 0.18** | **0.63** | **0.77 ± 0.18** | **0.34** |
| **hsa-miR-431** | **0.88 ± 0.17** | **0.60** | **0.57 ± 0.14** | **0.07** |
| **hsa-miR-125a-5p** | **0.88 ± 0.12** | **0.47** | **1.11 ± 0.04** | **0.07** |
| **sa-miR-191** | **0.88 ± 0.17** | **0.58** | **0.88 ± 0.08** | **0.27** |
| **hsa-miR-339-3p** | **0.88 ± 0.14** | **0.52** | **0.95 ± 0.07** | **0.61** |
| **hsa-miR-34a** | **0.87 ± 0.01** | **0.00** | **0.97 ± 0.08** | **0.76** |
| **hsa-miR-100** | **0.87 ± 0.06** | **0.17** | **0.86 ± 0.14** | **0.48** |
| **hsa-miR-146b-3p** | **0.87 ± 0.10** | **0.32** | **0.84 ± 0.33** | **0.71** |
| **hsa-miR-214** | **0.86 ± 0.09** | **0.27** | **0.78 ± 0.09** | **0.13** |
| **hsa-miR-196b** | **0.86 ± 0.19** | **0.59** | **0.88 ± 0.04** | **0.08** |
| **hsa-miR-455-5p** | **0.86 ± 0.06** | **0.15** | **0.86 ± 0.13** | **0.45** |
| **hsa-miR-320** | **0.86 ± 0.11** | **0.33** | **1.09 ± 0.05** | **0.20** |
| **hsa-miR-505** | **0.86 ± 0.02** | **0.00** | **0.72 ± 0.27** | **0.44** |
| **hsa-miR-433** | **0.85 ± 0.28** | **0.69** | **0.79 ± 0.16** | **0.36** |
| **hsa-miR-889** | **0.85 ± 0.13** | **0.40** | **0.39 ± 0.20** | **0.07** |
| **hsa-miR-20b** | **0.85 ± 0.13** | **0.41** | **0.87 ± 0.15** | **0.54** |
| **hsa-miR-140-3p** | **0.85 ± 0.04** | **0.04** | **0.77 ± 0.13** | **0.23** |
| **hsa-miR-491-5p** | **0.84 ± 0.29** | **0.68** | **0.94 ± 0.08** | **0.58** |
| **hsa-miR-197** | **0.84 ± 0.03** | **0.01** | **0.74 ± 0.04** | **0.01** |
| **hsa-miR-574-3p** | **0.84 ± 0.14** | **0.40** | **0.85 ± 0.07** | **0.14** |
| **hsa-miR-146b-5p** | **0.83 ± 0.01** | **0.00** | **0.81 ± 0.13** | **0.28** |
| **hsa-miR-193b** | **0.82 ± 0.12** | **0.31** | **0.86 ± 0.08** | **0.22** |
| **RNU44** | **0.82 ± 0.07** | **0.10** | **0.98 ± 0.13** | **0.90** |
| **hsa-miR-139-5p** | **0.81 ± 0.08** | **0.12** | **0.73 ± 0.03** | **0.00** |
| **hsa-miR-127-3p** | **0.81 ± 0.04** | **0.02** | **0.78 ± 0.07** | **0.07** |
| **hsa-miR-222** | **0.81 ± 0.07** | **0.09** | **0.88 ± 0.10** | **0.35** |
| **hsa-miR-107** | **0.79 ± 0.47** | **0.74** | **1.48 ± 0.64** | **0.58** |
| **hsa-miR-152** | **0.79 ± 0.09** | **0.12** | **0.86 ± 0.15** | **0.48** |
| **RNU48** | **0.78 ± 0.08** | **0.10** | **0.93 ± 0.06** | **0.43** |
| **hsa-miR-501-5p** | **0.78 ± 0.08** | **0.07** | **0.67 ± 0.12** | **0.08** |
| **hsa-miR-495** | **0.77 ± 0.06** | **0.03** | **0.59 ± 0.11** | **0.03** |
| **hsa-miR-215** | **0.76 ± 0.14** | **0.24** | **0.57 ± 0.21** | **0.18** |
| **hsa-miR-532-3p** | **0.76 ± 0.08** | **0.07** | **0.96 ± 0.12** | **0.79** |
| **hsa-miR-671-3p** | **0.76 ± 0.17** | **0.31** | **0.72 ± 0.11** | **0.11** |
| **hsa-miR-128** | **0.75 ± 0.13** | **0.18** | **0.82 ± 0.19** | **0.50** |
| **hsa-miR-485-3p** | **0.74 ± 0.11** | **0.12** | **0.40 ± 0.14** | **0.02** |
| **hsa-miR-30b** | **0.72 ± 0.10** | **0.07** | **0.76 ± 0.11** | **0.15** |
| **hsa-miR-379** | **0.72 ± 0.06** | **0.02** | **0.63 ± 0.06** | **0.01** |
| **hsa-miR-330-3p** | **0.72 ± 0.27** | **0.45** | **0.91 ± 0.19** | **0.72** |
| **hsa-miR-28-3p** | **0.71 ± 0.03** | **0.00** | **0.70 ± 0.08** | **0.04** |
| **hsa-miR-345** | **0.70 ± 0.12** | **0.11** | **0.49 ± 0.01** | **0.00** |
| **hsa-miR-370** | **0.68 ± 0.04** | **0.00** | **0.45 ± 0.07** | **0.00** |
| **hsa-miR-487b** | **0.67 ± 0.14** | **0.12** | **0.54 ± 0.04** | **0.00** |
| **hsa-miR-221** | **0.65 ± 0.08** | **0.02** | **0.59 ± 0.03** | **0.00** |
| **hsa-miR-30c** | **0.65 ± 0.10** | **0.05** | **0.67 ± 0.04** | **0.00** |
| **hsa-miR-134** | **0.63 ± 0.04** | **0.00** | **0.64 ± 0.04** | **0.00** |
| **hsa-miR-138** | **0.62 ± 0.38** | **0.45** | **1.22 ± 0.49** | **0.74** |
| **hsa-miR-503** | **0.59 ± 0.39** | **0.44** | **0.42 ± 0.11** | **0.01** |
| **hsa-miR-223** | **0.59 ± 0.10** | **0.03** | **0.62 ± 0.23** | **0.25** |
| **hsa-miR-217** | **0.59 ± 0.21** | **0.19** | **0.74 ± 0.09** | **0.07** |
| **hsa-miR-450b-5p** | **0.56 ± 0.12** | **0.04** | **0.54 ± 0.04** | **0.00** |
| **hsa-miR-597** | **0.50 ± 0.18** | **0.09** | **1.49 ± 0.68** | **0.59** |
| **hsa-miR-493** | **0.50 ± 0.09** | **0.01** | **0.67 ± 0.19** | **0.23** |
| **hsa-miR-200a** | **0.49 ± 0.16** | **0.06** | **0.87 ± 0.38** | **0.79** |
| **hsa-miR-187** | **0.46 ± 0.26** | **0.16** | **0.57 ± 0.14** | **0.06** |
| **hsa-miR-199a-5p** | **0.40 ± 0.15** | **0.27** | **11.16 ± 9.04** | **0.53** |
| **hsa-miR-149** | **0.34 ± 0.03** | **0.00** | **0.42 ± 0.12** | **0.02** |
| **hsa-miR-33b** | **0.24 ± 0.18** | **0.18** | **0.51 ± 0.40** | **0.76** |
| **hsa-miR-9** | **0.2 ± 0.04** | **0.00** | **0.35 ± 0.12** | **0.01** |
| **hsa-miR-517c** | **0.12 ± 0.07** | **0.02** | **0.14 ± 0.04** | **0.01** |
| **hsa-miR-139-3p** | **0.09 ± 0.04** | **0.00** | **0.07 ± 0.02** | **0.00** |
| **hsa-miR-1** | **N/A** |  | **N/A** |  |
| **hsa-miR-105** | **N/A** |  | **N/A** |  |
| **hsa-miR-122** | **N/A** |  | **N/A** |  |
| **hsa-miR-127-5p** | **N/A** |  | **N/A** |  |
| **hsa-miR-129-3p** | **N/A** |  | **N/A** |  |
| **hsa-miR-129-5p** | **N/A** |  | **N/A** |  |
| **hsa-miR-133b** | **N/A** |  | **N/A** |  |
| **hsa-miR-135a** | **N/A** |  | **N/A** |  |
| **hsa-miR-135b** | **N/A** |  | **N/A** |  |
| **hsa-miR-136** | **N/A** |  | **N/A** |  |
| **hsa-miR-141** | **N/A** |  | **N/A** |  |
| **hsa-miR-142-5p** | **N/A** |  | **N/A** |  |
| **hsa-miR-147** | **N/A** |  | **N/A** |  |
| **hsa-miR-147b** | **N/A** |  | **N/A** |  |
| **hsa-miR-153** | **N/A** |  | **N/A** |  |
| **hsa-miR-154** | **N/A** |  | **N/A** |  |
| **ath-miR159a** | **N/A** |  | **N/A** |  |
| **hsa-miR-182** | **N/A** |  | **N/A** |  |
| **hsa-miR-183** | **N/A** |  | **N/A** |  |
| **hsa-miR-184** | **N/A** |  | **N/A** |  |
| **hsa-miR-188-3p** | **N/A** |  | **N/A** |  |
| **hsa-miR-198** | **N/A** |  | **N/A** |  |
| **hsa-miR-205** | **N/A** |  | **N/A** |  |
| **hsa-miR-208** | **N/A** |  | **N/A** |  |
| **hsa-miR-208b** | **N/A** |  | **N/A** |  |
| **hsa-miR-211** | **N/A** |  | **N/A** |  |
| **hsa-miR-219-1-3p** | **N/A** |  | **N/A** |  |
| **hsa-miR-219-2-3p** | **N/A** |  | **N/A** |  |
| **hsa-miR-219-5p** | **N/A** |  | **N/A** |  |
| **hsa-miR-220** | **N/A** |  | **N/A** |  |
| **hsa-miR-220b** | **N/A** |  | **N/A** |  |
| **hsa-miR-220c** | **N/A** |  | **N/A** |  |
| **hsa-miR-296-3p** | **N/A** |  | **N/A** |  |
| **hsa-miR-298** | **N/A** |  | **N/A** |  |
| **hsa-miR-299-3p** | **N/A** |  | **N/A** |  |
| **hsa-miR-302a** | **N/A** |  | **N/A** |  |
| **hsa-miR-302b** | **N/A** |  | **N/A** |  |
| **hsa-miR-302c** | **N/A** |  | **N/A** |  |
| **hsa-miR-325** | **N/A** |  | **N/A** |  |
| **hsa-miR-329** | **N/A** |  | **N/A** |  |
| **hsa-miR-338-3p** | **N/A** |  | **2.68 ± 1.88** | **0.61** |
| **hsa-miR-34c-5p** | **N/A** |  | **N/A** |  |
| **hsa-miR-346** | **N/A** |  | **N/A** |  |
| **hsa-miR-363** | **N/A** |  | **N/A** |  |
| **hsa-miR-367** | **N/A** |  | **N/A** |  |
| **hsa-miR-369-3p** | **N/A** |  | **N/A** |  |
| **hsa-miR-371-3p** | **N/A** |  | **N/A** |  |
| **hsa-miR-372** | **N/A** |  | **N/A** |  |
| **hsa-miR-373** | **N/A** |  | **N/A** |  |
| **hsa-miR-375** | **N/A** |  | **N/A** |  |
| **hsa-miR-376b** | **N/A** |  | **N/A** |  |
| **hsa-miR-377** | **N/A** |  | **N/A** |  |
| **hsa-miR-380** | **N/A** |  | **N/A** |  |
| **hsa-miR-381** | **N/A** |  | **N/A** |  |
| **hsa-miR-383** | **N/A** |  | **N/A** |  |
| **hsa-miR-384** | **N/A** |  | **N/A** |  |
| **hsa-miR-412** | **N/A** |  | **N/A** |  |
| **hsa-miR-429** | **N/A** |  | **N/A** |  |
| **hsa-miR-448** | **N/A** |  | **N/A** |  |
| **hsa-miR-450a** | **N/A** |  | **N/A** |  |
| **hsa-miR-450b-3p** | **N/A** |  | **N/A** |  |
| **hsa-miR-453** | **N/A** |  | **N/A** |  |
| **hsa-miR-485-5p** | **N/A** |  | **N/A** |  |
| **hsa-miR-488** | **N/A** |  | **N/A** |  |
| **hsa-miR-490-3p** | **N/A** |  | **N/A** |  |
| **hsa-miR-491-3p** | **N/A** |  | **N/A** |  |
| **hsa-miR-492** | **N/A** |  | **N/A** |  |
| **hsa-miR-496** | **N/A** |  | **N/A** |  |
| **hsa-miR-499-3p** | **N/A** |  | **N/A** |  |
| **hsa-miR-499-5p** | **N/A** |  | **N/A** |  |
| **hsa-miR-501-3p** | **N/A** |  | **N/A** |  |
| **hsa-miR-504** | **N/A** |  | **N/A** |  |
| **hsa-miR-506** | **N/A** |  | **N/A** |  |
| **hsa-miR-507** | **N/A** |  | **N/A** |  |
| **hsa-miR-508-5p** | **N/A** |  | **N/A** |  |
| **hsa-miR-509-5p** | **N/A** |  | **N/A** |  |
| **hsa-miR-509-3-5p** | **N/A** |  | **N/A** |  |
| **hsa-miR-510** | **N/A** |  | **N/A** |  |
| **hsa-miR-511** | **N/A** |  | **N/A** |  |
| **hsa-miR-512-3p** | **N/A** |  | **N/A** |  |
| **hsa-miR-512-5p** | **N/A** |  | **N/A** |  |
| **hsa-miR-513-5p** | **N/A** |  | **N/A** |  |
| **hsa-miR-515-3p** | **N/A** |  | **N/A** |  |
| **hsa-miR-515-5p** | **N/A** |  | **N/A** |  |
| **hsa-miR-516a-5p** | **N/A** |  | **N/A** |  |
| **hsa-miR-516b** | **N/A** |  | **N/A** |  |
| **hsa-miR-517a** | **N/A** |  | **N/A** |  |
| **hsa-miR-517b** | **N/A** |  | **N/A** |  |
| **hsa-miR-518a-3p** | **N/A** |  | **N/A** |  |
| **hsa-miR-518a-5p** | **N/A** |  | **N/A** |  |
| **hsa-miR-518c** | **N/A** |  | **N/A** |  |
| **hsa-miR-518d-3p** | **N/A** |  | **N/A** |  |
| **hsa-miR-518d-5p** | **N/A** |  | **N/A** |  |
| **hsa-miR-518f** | **N/A** |  | **N/A** |  |
| **hsa-miR-519c-3p** | **N/A** |  | **N/A** |  |
| **hsa-miR-519d** | **N/A** |  | **N/A** |  |
| **hsa-miR-519e** | **N/A** |  | **N/A** |  |
| **hsa-miR-520a-3p** | **N/A** |  | **N/A** |  |
| **hsa-miR-520a-5p** | **N/A** |  | **N/A** |  |
| **hsa-miR-520b** | **N/A** |  | **N/A** |  |
| **hsa-miR-520d-5p** | **N/A** |  | **N/A** |  |
| **hsa-miR-520e** | **N/A** |  | **N/A** |  |
| **hsa-miR-520f** | **N/A** |  | **N/A** |  |
| **hsa-miR-520g** | **N/A** |  | **N/A** |  |
| **hsa-miR-521** | **N/A** |  | **N/A** |  |
| **hsa-miR-522** | **N/A** |  | **N/A** |  |
| **hsa-miR-524-5p** | **N/A** |  | **N/A** |  |
| **hsa-miR-525-3p** | **N/A** |  | **N/A** |  |
| **hsa-miR-525-5p** | **N/A** |  | **N/A** |  |
| **hsa-miR-526b** | **N/A** |  | **N/A** |  |
| **hsa-miR-541** | **N/A** |  | **N/A** |  |
| **hsa-miR-544** | **N/A** |  | **N/A** |  |
| **hsa-miR-548a-3p** | **N/A** |  | **N/A** |  |
| **hsa-miR-548a-5p** | **N/A** |  | **N/A** |  |
| **hsa-miR-548b-3p** | **N/A** |  | **N/A** |  |
| **hsa-miR-548c-3p** | **N/A** |  | **N/A** |  |
| **hsa-miR-548c-5p** | **N/A** |  | **N/A** |  |
| **hsa-miR-548d-3p** | **N/A** |  | **N/A** |  |
| **hsa-miR-548d-5p** | **N/A** |  | **N/A** |  |
| **hsa-miR-551b** | **N/A** |  | **N/A** |  |
| **hsa-miR-556-3p** | **N/A** |  | **N/A** |  |
| **hsa-miR-556-5p** | **N/A** |  | **N/A** |  |
| **hsa-miR-561** | **N/A** |  | **N/A** |  |
| **hsa-miR-570** | **N/A** |  | **N/A** |  |
| **hsa-miR-576-5p** | **N/A** |  | **N/A** |  |
| **hsa-miR-582-3p** | **N/A** |  | **N/A** |  |
| **hsa-miR-589** | **N/A** |  | **N/A** |  |
| **hsa-miR-615-3p** | **N/A** |  | **N/A** |  |
| **hsa-miR-615-5p** | **N/A** |  | **N/A** |  |
| **hsa-miR-616** | **N/A** |  | **N/A** |  |
| **hsa-miR-618** | **N/A** |  | **N/A** |  |
| **hsa-miR-624** | **N/A** |  | **N/A** |  |
| **hsa-miR-627** | **N/A** |  | **N/A** |  |
| **hsa-miR-642** | **N/A** |  | **N/A** |  |
| **hsa-miR-651** | **N/A** |  | **N/A** |  |
| **hsa-miR-653** | **N/A** |  | **N/A** |  |
| **hsa-miR-654-3p** | **N/A** |  | **N/A** |  |
| **hsa-miR-672** | **N/A** |  | **N/A** |  |
| **hsa-miR-674** | **N/A** |  | **N/A** |  |
| **hsa-miR-708** | **N/A** |  | **N/A** |  |
| **hsa-miR-871** | **N/A** |  | **N/A** |  |
| **hsa-miR-872** | **N/A** |  | **N/A** |  |
| **hsa-miR-873** | **N/A** |  | **N/A** |  |
| **hsa-miR-875-3p** | **N/A** |  | **N/A** |  |
| **hsa-miR-876-3p** | **N/A** |  | **N/A** |  |
| **hsa-miR-876-5p** | **N/A** |  | **N/A** |  |
| **hsa-miR-885-3p** | **N/A** |  | **N/A** |  |
| **hsa-miR-888** | **N/A** |  | **N/A** |  |
| **hsa-miR-890** | **N/A** |  | **N/A** |  |
| **hsa-miR-891a** | **N/A** |  | **N/A** |  |
| **hsa-miR-891b** | **N/A** |  | **N/A** |  |
| **hsa-miR-892a** | **N/A** |  | **N/A** |  |
| **hsa-miR-96** | **N/A** |  | **N/A** |  |

ECs were kept under static conditions as controls (CL) or exposed to OS (0.5±4 dynes/cm2) or PS (12±4 dynes/cm2) for 24 h, and their total RNAs were collected and subjected to miR microarray analysis. Data are mean±SEM from three independent experiments. N/A: Not available, indicating that at least one data are not available for three independent experiments. *: A mean OS/CL ratio ≥ 1.2 and *p*≤0.05. **: A mean PS/CL ratio > 1.2 and *p*≤0.05.
