# Supplementary material for: Mechanoresponsive Smad5 Enhances MiR-487a Processing to Promote Vascular Endothelial Proliferation in Response to Disturbed Flow
Source: Front Cell Dev Biol. 2021 Apr 20;9:647714. doi: 10.3389/fcell.2021.647714 (PMC8093806; doi:10.3389/fcell.2021.647714)
Supplement: Supplementary file 2 [file Table_2.DOCX]

**Online Table S2. Primer sequences used for the qRT-PCR analysis**

| Application | Oligonucleotides | Sequences(5’ → 3’) |
| --- | --- | --- |
| Mature miR-487a | RT | GTTGGCTCTGGTGCAGGGTCCGAGGTATTCGCACCAGAGCCAACAACTGG |
|  | Forward | GCGCAATCATACAGGGACAT |
|  | Reverse | GTGCAGGGTCCGAGGT |
| Primary miR-487a | Forward | CTCGTGAAATACTCGTAAGGATGA |
|  | Reverse | GGCCACAGCATGTGAGTCT |
| CBP 3’UTR | Forward | GGGTGGATTGATGTTTAAAGAAA |
|  | Reverse | CCCCAAACAAAAACAAAACG |
| P53 3’UTR | Forward | TCCACTTCTTGTTCCCCACT |
|  | Reverse | AAAGACCCAAAACCCAAAATG |
