# Supplementary material for: Mechanoresponsive Smad5 Enhances MiR-487a Processing to Promote Vascular Endothelial Proliferation in Response to Disturbed Flow
Source: Front Cell Dev Biol. 2021 Apr 20;9:647714. doi: 10.3389/fcell.2021.647714 (PMC8093806; doi:10.3389/fcell.2021.647714)
Supplement: Supplementary file 3 [file Data_Sheet_1.DOCX]

**ONLINE SUPPLEMENTAL DOCUMENT**

**ONLINE METHODS**

**Materials.** Rabbit polyclonal antibodies (pAbs) against phospho-Smad5 and Smad5 were obtained from Abcam (Cambridge, UK). Anti-histone H1 mouse monoclonal antibody (mAb) was purchased from Millipore (Temecula, CA). Rabbit mAb against Drosha, rabbit pAbs against phospho-Rb and acetyl-p53 and mouse mAbs against p53, cyclin A were purchased from Cell Signaling Technology (Beverly, MA). Anti-Rb and anti-CBP rabbit pAbs were obtained from Santa Cruz Biotechnology (Santa Cruz, CA). All other chemicals of reagent grade were obtained from Sigma (St. Louis, MO).

**Cell cultures.** Human aortic ECs were obtained commercially (Clonetics, Palo Alto, CA) ( Tsai et al., 2009). Cells were resuspended in a culture medium consisting of Medium 199 ( M199, Gibco, Grand Island, NY) supplemented with 10% fetal bovine serum (FBS, Gibco) and 1% penicillin/streptomycin (Gibco). ECs were seeded onto glass slides (Corning, New York) pre-coated with fibronectin, and then kept under static condition or subjected to different flow conditions. BAECs were obtained from Clonetics and cultured in DMEM (Gibco) with 10% FBS and 1% penicillin/streptomycin, and used in some transfection experiments.

**Shear stress experiment.** Cultured ECs were subjected to PS at 12 ± 4 dynes/cm^2^ or OS at 0.5 ± 4 dynes/cm^2^ in a parallel-plate flow chamber, as previously described (Lee et al., 2017). In brief, the flow channel in the chamber was created by sandwiching a silicon gasket with 25 mm in width (w), 50 mm in length, and 0.25 mm in height (h), between the cell-seeded glass slide and a polycarbonate base plate on top of a stainless plate. The channel was connected to a perfusion loop system, which was kept at 37°C in a constant-temperature controlled enclosure, with pH maintained at 7.4 by continuous gassing with humidified 5% CO_2_ in air. The oscillatory flow is composed of a low level of mean flow (shear stress = 0.5 dyne/cm^2^) supplied by a hydrostatic flow system to provide the basal nutrient and oxygen delivery, and a superimposed sinusoidal oscillation using a piston pump with a frequency of 1 Hz and a peak-to peak amplitude of ± 4 dynes/cm^2^. The PS has a high level of mean shear stress at 12 dynes/cm^2^ plus the superimposion of oscillation with a frequency of 1 Hz and an amplitude of ± 4 dynes/cm^2^.

**Knockdown and overexpression of miR-487a, CBP, and p53.** Knockdown of miR-487a expression in ECs was performed with the miR-487a inhibitor (anti-miR-487a) (Ambion, Austin, TX). The overexpression of miR-487a was achieved by using miR-487a mimic (pre-miR-487a) (Ambion). For knockdown of CBP and p53, the specific siRNAs of CBP and p53 purchased from Invitrogen (Carlsbad, CA) were used. Transfection procedures were performed with RNAiMAX kit (Invitrogen), according to the manufacturer’s standard protocols.

**Quantitative real-time PCR analysis of miR expression.** After exposure to shear flow, total RNA was extracted by Trizol reagent (Invitrogen). Expression of mature miR-487a was assessed by two-step quantitative real-time PCR by using the miR-specific primers. The design of the primers of pri-miR-487a and mature miR-487a and the designated molecules was provided in the online Table S2.

**RNA-immunoprecipitation (IP) assay.** ECs subjected to shear stress or transfected with control miR or pre-miR-487a were lysed in lysis buffer containing 25 mmol/L Tris-HCl, pH 7.4, 150 mmol/L KCl, 0.5% NP-40, 2 mmol/L EDTA, 1 mmol/L NaF, RNase inhibitor 100 U/mL, 0.5 mmol/L DTT, and protease inhibitor cocktail. After centrifugation by 13,000 rpm at 4°C, the supernatant was collected and subjected to immunoprecipitation with anti-AGO2 specific antibody. The RNA was isolated using Trizol reagent following a standard protocol. The purified RNA was analyzed by quantitative real-time RT-PCR.

**Primers for RNA-IP.** Human pri-miR-487a: 5’-ctcgtgaaatactcgtaaggatga-3’ and 5’-ggccacagcatgtgagtct-3’. CBP: 5’-gggtggattgatgtttaaagaaa-3’ and 5’-ccccaaacaaaaacaaaacg-3’. p53: 5’-tccacttcttgttccccact-3’ and 5’-aaagacccaaaacccaaaatg-3’ (online Table S2)

**Diseased human coronary arteries.** Diseased human coronary arteries and control internal thoracic arteries were obtained from patients (n=7) with end-stage heart failure undergoing heart transplantation at the Tri-Service General Hospital, as approved by the Hospital Human Subjects Review Committee and Ethics Review Board of the National Health Research Institutes (Number: EC1020901-E). These diseased human coronary arteries contained various stages of atherosclerosis from initial neointima lesions without macrophages, mild lesions with fatty streaks and macrophages to advanced lesions with fibrotic cap and calcification, according to American Heart Association Guidelines for histological classification of atherosclerotic lesions (Stary et al., 1995). The samples were fixed and embedded in paraffin blocks, as previously described (Stary et al., 1995). Cross-sections of the affected arteries were examined by *in situ* hybridization for miR-487a expression and immunostaining for EC marker vWF.

**Animal model of aortic stenosis.** For aortic stenosis experiments, a U-shaped titanium clip (Ethicon Endo-Surgery) was surgically applied to the rat abdominal aorta, as previously described (Miao et al., 2005). In brief, after anesthetization with isoflurane, a segment of the rat abdominal aorta (between renal and iliac arteries) was exposed, and a clip was placed around the isolated segment (~ 1 cm proximal from the iliac arterial bifurcation), which resulted in a 65% construction of the aorta diameter detected by ultrasonography (Zhou et al., 2012). The experimental rats were intraarterially injected with anti-miR-487a mixed with invivofectamin (Invitrogen) following the manufacturer’s protocol; the control rats received control miR. The rat was deeply euthanized with CO_2_ at 7 day after surgery, and the aorta was intracardially perfusion-fixed for 15 min with 300 mL 10 % neutral-buffered zinc-formalin at 120 mm Hg. The fixed aorta was subjected to *en face* staining. The animal experiments were performed in accordance with National Institutes of Health guidelines and with the approval of the Animal Research Committee of National Health Research Institutes.

***En Face* staining.** The formalin-fixed rat aorta tissues were washed with Tris-buffered saline (TBS), and the adventitia was carefully removed. The aorta was longitudinally dissected with microdissecting scissors and pinned flatly for *en face* staining, as previously described (de Planell-Saguer et al., 2010). In brief, the luminal surface of the aorta was immediately permeabilized with 0.5% Triton X-100 in PBS for 15 min, and then washed with PBS. The tissues were pre-hybridized (3% BSA in 4X SSC) at 40°C for 20 min, followed by hybridization with Dig-conjugated locked nucleic acid (LNA)-miR-487a probe (EXIQON, Vedbaek, Denmark) dissolved in hybridization buffer (10% dextran sulfate in 4X SSC) at 40°C for 90 min, and then washed at 45°C with wash buffers I, II, and III. The tissues were covered with 3% hydrogen peroxide solution for 20 min and then washed with TN buffer (0.1 mol/L Tris-HCl, pH 7.5, 0.15 mol/L NaCl). The tissues were blocked with 1% BSA for 30 min, stained with anti-rabbit VWF antibody for 1 h at room temperature, washed with PBS, and blocked by TNB blocking buffer [0.1 mol/L Tris-HCL, pH 7.5, 0.15 mol/L NaCl, 0.5% (wt/vol) blocking reagent (4% BSA in 1X PBS)] for 30 min. The tissues were incubated with anti-DIG antibody in TNB blocking solution overnight at 4°C, and then washed with TNT buffer (0.1 mol/L Tris-HCL, pH 7.5, 0.15 mol/L NaCl, 0.2% (vol/vol) Triton X-100), followed by incubation with TSA and Alexa Fluor 488-conjugated goat anti-rabbit IgG (Invitrogen). The samples were counterstained with DAPI to show cell nuclei and photographed under a Leica TCS SP5 confocal microscope.

**Western blot analysis.** ECs were lysed with a buffer containing 1% NP-40, 0.5% sodium deoxycholate, 0.1% SDS, protease and phosphatase inhibitor cocktails. The total cell lysate was separated by SDS-PAGE and transferred onto a nitrocellular membrane, The protein was detected by the indicated primary antibodies, secondary HRP-conjugated antibodies, and ECL reagent was used for reacting with HRP to display chemiluminescent signal.

**RNA-Electrophoretic mobility shift assay (EMSA).** RNA-EMSA was conducted using *in vitro* transcribed bio-UTP-labeled pri-miR-487a probe (~150 nt). The pri-miR-487a probe was heat-denatured for 6 min at 65°C and renatured prior to being added to the binding buffer containing nuclear proteins (10 μg) of ECs stimulated by shear stress. The binding reaction was performed at 4°C for 50 min in EMSA buffer (20 mmol/L Hepes-KOH, pH 7.5, 12 mmol/L MgCl_2_, 60 mmol/L KCl, 20% glycerol, and 4 mmol/L DTT). 0.1 μg poly[dI-dC], 2.5 μg yeast tRNA, 0.1 μg BSA and RNAse inhibitor (Invitrogen) were added to each 20 μL reaction mixture. The bound complexes were resolved on native 4% polyacrylamide gel in 0.5X TB buffer (45 mmol/L Tris-Borate). The biotin-labeled RNAs were transferred to the positively-charged nylon membrane and detected by using the Chemiluminescent EMSA kit (Pierce, Rockford, IL).

**Pri-miR-487a overexpression construct.** The pcDNA3.1(+)-miR-487a-construct contains 522-bp human miR-487a genomic fragments, which is cloned from HEK293T cells by using the designated primers (5’-CCGGGATCCTCTTGCTGGGGAAGCTTTCTG-3’ and 5’-CGG AATTCCACTGGTCAACTTCCCCCAAT-3’) and inserted into a pcDNA3.1(+) vector (Invitrogen) by BamHⅠand EcoRⅠ. The 159 bp wild type pri-miR-487a sequence was synthesized by PCR from pcDNA3.1(+)-miR-487a-522 bp using primers: 5’-CCGGGATCC GACTCTGGTGAAGACGTGGG-3’ and 5’-CGGAATTCGGGACGCTGTCCGGGAA-3’. This wild type product was used for two step PCR mutagenesis to create the miR-487a mutants, as previously described (Davis et al., 2010). The mutagenesis primer is 5’-AATCATAAAAGGACA TCCAGTTTTTCAGT-3’. Both of wild type and mutant pri-miR487a-159 bp were inserted into pcDNA3.1(+) vector by BamHⅠand EcoRⅠ.

***In vitro* pri-miRNA processing assays.** *In vitro* pri-miRNA processing assay was performed, as previously described (Guil et al., 2007). In brief, the biotin-labeled 522-nucleotide pri-miR-487a was prepared by *in vitro* transcription with T7 RNA polymerase in the presence of biotin-UTP, using human miR-487a gene cloned into pcDNA3.1(+) vector as a template. ECs were subjected to different flow patterns and the nuclear fraction was obtained from the crude extract by using a HEPES buffer (10 mmol/L HEPES, pH 7.9, 85 mmol/L KCl, 1 mmol/L EDTA, 1 mmol/L DTT, 0.5% NP-40, protease and phosphatase inhibitor cocktails). Thirty μg of nuclear extract were mixed with a reaction buffer (6.4 mmol/L MgCL_2_), biotin-labeled pri-miR-487a, and RNase inhibitor in RNase-free water, and then incubated at 37°C for 90 min. The reaction mixtures were subjected to phenol-chroloform extraction, precipitation, and gel electrophoresis with 12.5% urea-polyacrylamide, followed by positively-charged nylon membrane transfer and detection by using the Chemiluminescent EMSA kit (Pierce).

***In vitro* transcription.** PcDNA3.1-miR-487a plasmid DNA that linearized with XhoI and ethanol purified was used as a template for *in vitro* transcription with MAXIscript kit (ambion).

**Generation of luciferase reporter construct and luciferase reporter assay.** To generate reporter vectors bearing miR-487a binding sites and wild-type and mutant 3’UTR of CBP and p53, the sense and antisense strands of oligonucleotides bearing predicted miR-487a binding sequences were commercially synthesized, annealed, and cloned into HindIII and MluI of the pMIR-REPORT luciferase vector (Ambion). These sense and antisense oligonucleotides were designed as follows: miR-487a binding site: 5’-AGCTAACTGGATGTCCCTGTATGATT-3’ and 5’-CGCGAATCATACAGGGACATCCAGTT-3’; Wild type CBP 3’UTR: 5’-AGCTGTG GGCGTCTCCCAGTATTACCCTGGATGATAGGAATT-3’ and 5’-CGCGAATTCCTATCAT CCAGGGTAATACTGGGAGACGCCCAC-3’. Mutant CBP 3’UTR: 5’-AGCTGTGGGCGT CTCCCAGTATTACCCTGGGCAGTAGGAATT-3’ and 5’-CGCGAATTCCTACTGCCCAG GGTAATACTGGGAGACGCCCAC-3’. Wild type p53 3’UTR: 5’-AGCTTGGAGGATTTC ATCTCTTGTATATGATGTGGATCCACCAAGA-3’ and 5’-CGCGTCTTGGTGGATCCAC ATCATATACAAGAGATGAAATCCTCCA-3’. Mutant p53 3’UTR: 5’-AGCTTGGAGGAT TTCATCTCTTGTACGCAGCGTGGATCCACCAAGA-3’ and 5’-CGCGTCTTGGTGGATC CACGCTGCGTACAAGAGATGAAATCCTCCA-3’). For luciferase assay, the pSV-β- galactosidase plasmid was co-transfected with the luciferase reporter vectors to normalize the transfection efficiency. Twenty-four hours after transfection, luciferase activity was measured by using the Luciferase assay system (Promega) and normalized to the β-galactosidase activity assessed by using o-nitrophenyl-β-D-galactopyranoside.

**BrdU incorporation assay.** ECs were transfected with 1 nmol/L anti-miR or pre-miR and then cultured on glasses coated with fibronetin. The cells were pre-treated with 32.5 μmol/L BrdU for 30 min and then subjected to PS or OS with flow medium containing BrdU for 12 h. The cells were fixed with 4% paraformaldehyde. After incubation with NaBH4, 2N HCl, and sodium tetraborate, the cells were stained with FITC-conjugated anti-BrdU antibody, with nuclear counterstaining by propidium iodide. The stained samples were examined using an inverted microscope (Axiovert 200M; Zeiss) with a 20X objective. Proliferation was assessed based on the percentage of nuclei exhibiting BrdU incorporation.

**Statistic analysis.** Results are expressed as mean ± SEM. Statistical significance was determined using the Mann-Whitney rank sum test in the program SigmaStat 3.5 for two groups of data and a one-way analysis of variance (ANOVA), followed by the Scheffe’s test for multiple comparisons. The level of statistical significance was defined as *p*<0.05 from 3-5 separate experiments.

**ONLINE REFERENCES**

Davis B.N., Hilyard A.C., Nguyen P.H., Lagna G., and Hata A. (2010) Smad proteins bind a conserved RNA sequence to promote microRNA maturation by Drosha. *Mol. Cell* 39, 373-384. doi: 10.1016/j.molcel.2010.07.011

de Planell-Saguer M., Rodicio M.C., and Mourelatos Z. (2010) Rapid in situ codetection of noncoding RNAs and proteins in cells and formalin-fixed paraffin-embedded tissue sections without protease treatment. *Nat. Protoc.* 5, 1061-1073. doi: 10.1038/nprot.2010.62

Guil S., and Caceres J.F. (2007) The multifunctional RNA-binding protein hnRNP A1 is required for processing of miR-18a. *Nat. Struct. Mol. Biol.* 14, 591-596. doi: 10.1038/nsmb1250

Lee D.Y., Lin T.E., Lee C.I., Zhou J., Huang Y.H., Lee P.L., et al.(2017) MicroRNA-10a is crucial for endothelial response to diﬀerent ﬂow patterns via interaction of retinoid acid receptors and histone deacetylases, Proc. Natl. Acad. Sci. U.S.A. 114, 2072–2077. doi: 10.1073/pnas.1621425114

Miao H., Hu Y.L., Shiu Y.T., Yuan S., Zhao Y., Kaunas R., et al. (2005) Effects of flow patterns on the localization and expression of VE-cadherin at vascular endothelial cell junctions: in vivo and in vitro investigations. *J Vasc Res.* 42, 77-89. doi: 10.1159/000083094

Stary H.C., Chandler A.B., Dinsmore R.E., Fuster V., Glagov S., Insull W., et al. (1995) A definition of advanced types of atherosclerotic lesions and a histological classification of atherosclerosis. A report from the Committee on Vascular Lesions of the Council on Arteriosclerosis, American Heart Association. *Circulation.* 92, 1355-1374. doi: 10.1161/01.cir.92.5.1355.

Tsai M.C., Chen L., Zhou J., Tang Z., Hsu T.F., Wang Y., et al. (2009). Shear stress induces synthetic-to-contractile phenotypic modulation in smooth muscle cells via peroxisome proliferator-activated receptor alpha/delta activations by prostacyclin released by sheared endothelial cells. *Circ Res.* 105, 471-480. doi: 10.1161/CIRCRESAHA.109.193656

Zhou J., Lee P.L., Tsai C.S., Lee C.I., Yang T.L., Chuang H.S., et al. (2012) Force-specific activation of Smad1/5 regulates vascular endothelial cell cycle progression in response to disturbed flow. *Proc. Natl. Acad. Sci. U.S.A.* 109, 7770-7775. doi: 10.1073/pnas.1205476109
